# Crystal structure and Hirshfeld surface analysis of 5-acetyl-3-amino-6-methyl-*N*-phenyl-4-[(*E*)-2-phenyl­ethen­yl]thieno[2,3-*b*]pyridine-2-carbox­amide

**DOI:** 10.1107/S2056989022000743

**Published:** 2022-01-28

**Authors:** Shaaban K. Mohamed, Etify A. Bakhite, Sevim Türktekin Çelikesir, Hajjaj H. M. Abdu-Allah, Mehmet Akkurt, Omaima F. Ibrahim, Joel T. Mague, Safiyyah A. H. Al-Waleedy

**Affiliations:** a Chemistry and Environmental Division, Manchester Metropolitan University, Manchester, M1 5GD, England; b Chemistry Department, Faculty of Science, Minia University, 61519 El-Minia, Egypt; c Chemistry Department, Faculty of Science, Assiut University, 71516 Assiut, Egypt; d Department of Physics, Faculty of Sciences, Erciyes University, 38039 Kayseri, Turkey; e Pharmaceutical Chemistry Department, Faculty of Pharmacy, Assiut University, Assiut, Egypt; f Department of Chemistry, Tulane University, New Orleans, LA 70118, USA; g Department of Chemistry, Faculty of Science, Taiz University, Taiz, Yemen

**Keywords:** crystal structure, styryl group, thio­pyridine, *N*-phenyl­carboxamido group, C–H⋯π(ring) inter­actions, Hirshfeld surface analysis

## Abstract

The asymmetric unit of the title compound, C_25_H_21_N_3_O_2_S, comprises four mol­ecules. Their conformations differ primarily in the orientations of the styryl and the *N*-phenyl­carboxamido groups. In the crystal, inter­molecular N—H⋯N, C—H⋯O and C—H⋯S hydrogen-bonding contacts as well a C—H⋯π(ring) inter­actions lead to the formation of a layer structure parallel to (010). Hirshfeld surface analysis revealed that H⋯H inter­actions represent the main contributions to the crystal packing.

## Abstract 2

The asymmetric unit of the title mol­ecule consists of four mol­ecules that differ primarily in the orientations of the styryl and the *N*-phenyl­carboxamido groups.

## Chemical context

Thieno­pyridine derivatives are well known to possess various functional and medicinal properties with general applications as synthetic building blocks or as pharmaceuticals (Litvinov *et al.*, 2005 ▸; Dotsenko *et al.*, 2020 ▸; Bakhite, 2003 ▸; Al-Waleedy *et al.*, 2020 ▸; Abuelhassan *et al.*, 2021 ▸). Many thieno­pyridines are reported to show anti­cancer (Zeng *et al.*, 2010 ▸), anti­parasitic (Bernardino *et al.*, 2006 ▸), insecticidal (El-Dean *et al.*, 2019 ▸), anti­microbial (Abdel-Rahman *et al.*, 2003 ▸; Eldin, 1999 ▸) and anti­diabetic (Bahekar *et al.*, 2007 ▸) activities. Encouraged by the above facts, we report in this communication the synthesis and crystal structure determination of the title compound, C_25_H_21_N_3_O_2_S (**I**). 
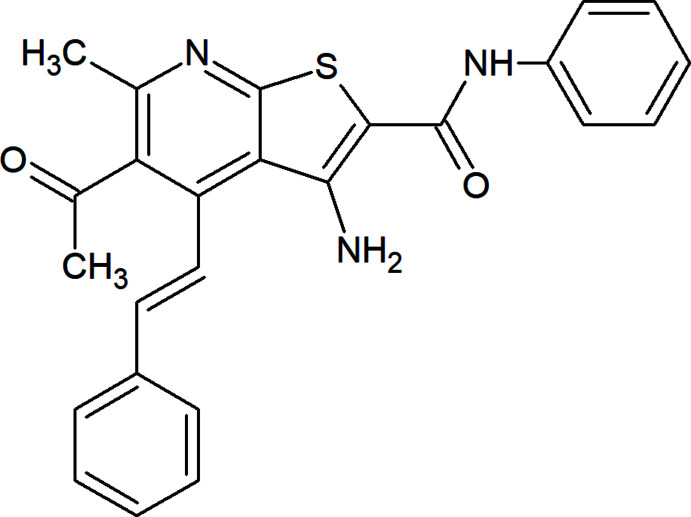


## Structural commentary

**Figure 1:**
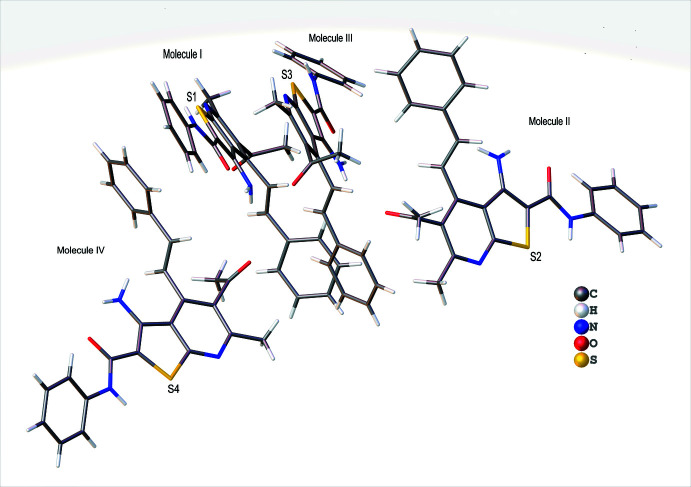
The four mol­ecules (I, II, III and IV) in the asymmetric unit of (**I**).

**Figure 2:**
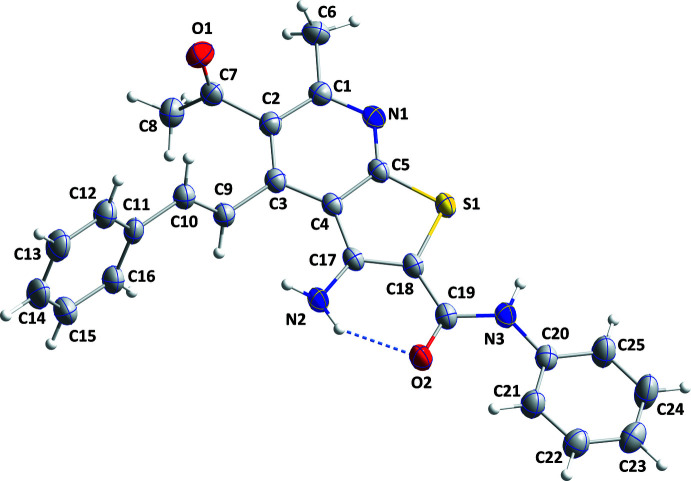
Mol­ecule I with displacement ellipsoids for the non-hydrogen atoms drawn at the 30% probability level. The intra­molecular N—H⋯O hydrogen bond is depicted by a dashed line.

**Figure 3:**
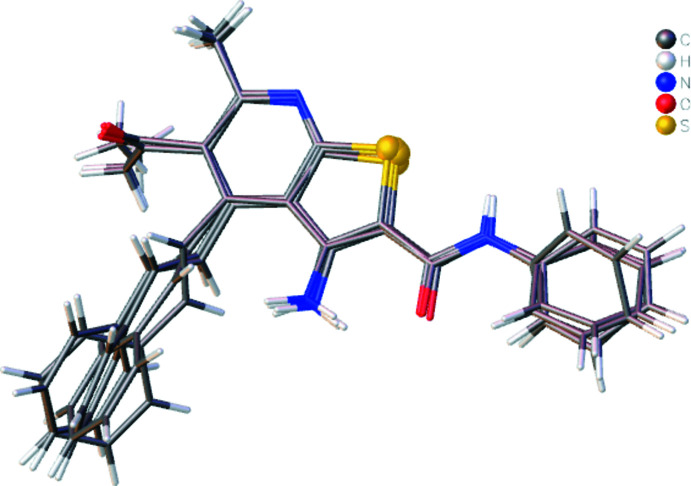
Overlay image of the four mol­ecules (I, II, III and IV) in the asymmetric unit of the title compound.

The asymmetric unit of (**I**) contains four mol­ecules (Fig. 1 ▸) of which one (mol­ecule I) is represented in an *ORTEP*-style plot in Fig. 2 ▸. The conformational differences between mol­ecules I, II, III and IV are highlighted in the overlay diagram shown in Fig. 3 ▸. The maximum r.m.s. deviation of the overlay between mol­ecules I, II, III and IV is 0.498 Å. The conformations of the four mol­ecules differ primarily in the varying orientations of the styryl and the *N*-phenyl­carboxamido groups, as indicated by the torsion and dihedral angles collated in Tables 1 ▸ and 2 ▸. The orientations of the latter substituents are partially determined by the intra­molecular N—H⋯O hydrogen bond (Table 3 ▸ and Fig. 2 ▸). In each mol­ecule, both the thio­phene and pyridine rings are planar to within 0.0235 (11) Å (maximum r.m.s deviation = 0.0163 Å) and 0.0197 (12) Å (maximum r.m.s deviation = 0.0125 Å). Other bond lengths and angles are all in the expected ranges.

## Supra­molecular features

**Figure 4:**
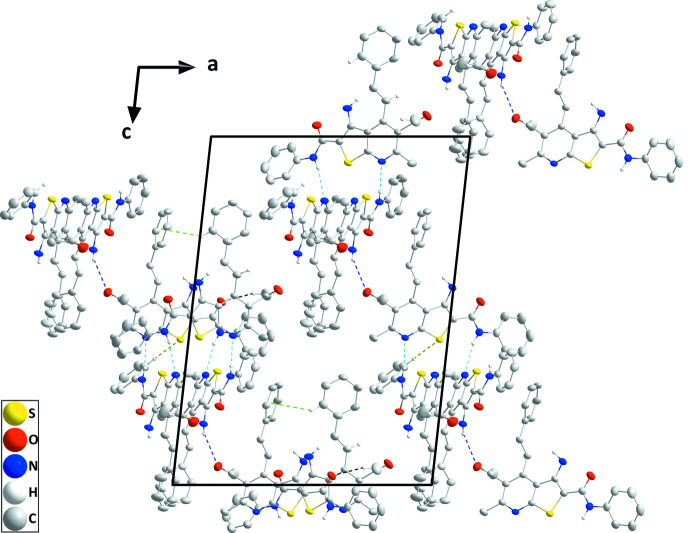
Packing in the crystal of (**I**) viewed along the *b* axis direction. N—H⋯O, C—H⋯O, N—H⋯N and C—H⋯S hydrogen bonds are depicted, respectively, by dark blue, black, light blue and yellow dashed lines. The C—H⋯π(ring) inter­actions are illustrated by green dashed lines.

In the crystal, various hydrogen-bonding inter­actions are found (Table 3 ▸). The strongest stem from inter­actions between the amide NH group and the pyridine N atom of a neighbouring mol­ecule (N3—H3*A*⋯N4^i^, N6—H6*D*⋯N7^ii^, N9—H9*A*⋯N10^v^ and N12—H12*A*⋯N1^vi^). Weaker C33—H33*C*⋯O8^iii^ and C75—H75⋯S4^v^ inter­actions between a methyl group and an a carbonyl O atom, and between a phenyl CH group and a thio­phene S atom, respectively, consolidate the packing. Together with three sets of C—H⋯π(ring) inter­actions, supra­molecular layers parallel to the *ac* plane with a width corresponding to *b*/2 are formed (Figs. 4 ▸ and 5 ▸).

## Hirshfeld surface analysis

**Figure 6:**
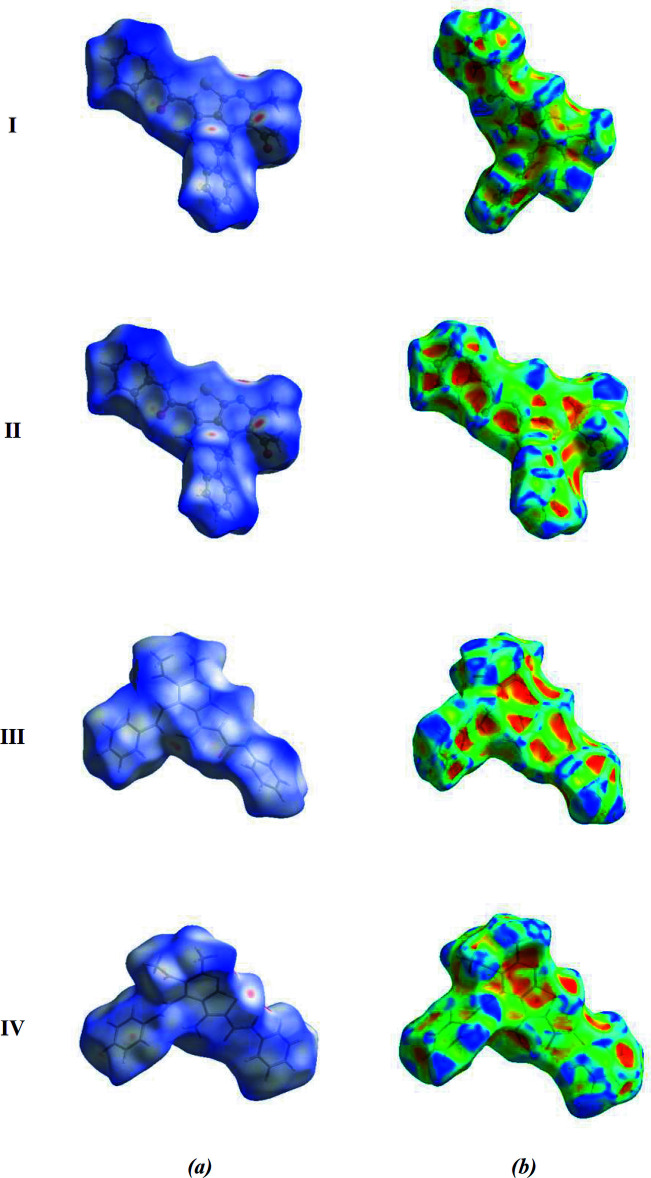
A view of the three-dimensional Hirshfeld surface for the four mol­ecules (I, II, III and IV) in the asymmetric unit of the title compound, plotted over (*a*) *d*_norm_ and (*b*) shape-index.

**Figure 7:**
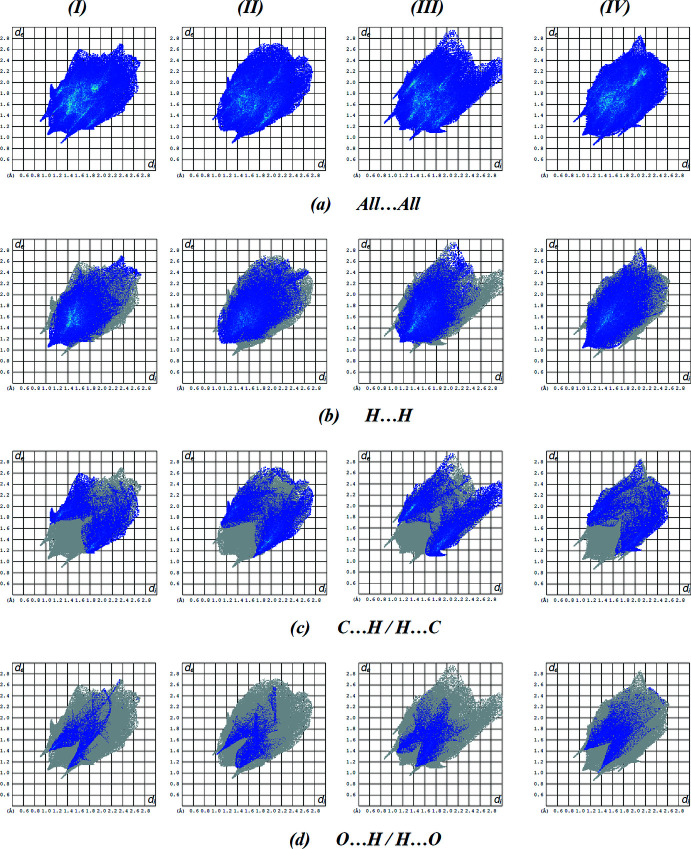
A view of the two-dimensional fingerprint plots for the four mol­ecules (I, II, III and IV) in the asymmetric unit of the title compound, showing (*a*) all inter­actions, and delineated into (*b*) H⋯H, (*c*) C⋯H/H⋯C and (*d*) O⋯H/H⋯O inter­actions. The *d*_i_ and *d*_e_ values are the closest inter­nal and external distances (in Å) from given points on the Hirshfeld surface.

For the four mol­ecules I, II, III and IV, inter­molecular inter­actions (Table 4 ▸) were qu­anti­fied using Hirshfeld surface analysis and the associated two-dimensional fingerprint plots generated. The calculations and visualization were carried out using *Crystal Explorer 17.5* (Turner *et al.*, 2017 ▸). Fig. 6 ▸ shows the Hirshfeld surface of the four mol­ecules in (**I**) mapped over *d*_norm_ in a fixed colour scale of −0.3297 (red) to + 1.5167 (blue) a.u. for mol­ecule I, −0.3246 (red) to +1.4683 (blue) a.u. for mol­ecule II, −0.3890 (red) to +2.0338 (blue) a.u. for mol­ecule III, and −0.3870 (red) to +1.8555 (blue) a.u. for mol­ecule IV. The red spots on the Hirshfeld surface are indicative of contacts shorter than van der Waals separations and represent N—H⋯N, N—H⋯O, C—H⋯O and C—H⋯S contacts. Fig. 7 ▸ displays the full two-dimensional fingerprint plot and those delineated into the major contacts. H⋯H inter­actions (46.5% contribution for I; 47.0% for II; 44.7% for III; 45.5% for IV) are the major factor in the crystal packing with C⋯H/H⋯C (22.7% for I; 27.9% for II; 28.1% for III; 20.2% for IV) and O⋯H/H⋯O (9.7% for I; 8.9% for II; 11.3% for III; 12.6% for IV) inter­actions representing the next highest contributions. The percentage contributions of other weak inter­actions are listed in Table 5 ▸.

The fact that the same inter­actions result in different contributions to the Hirshfeld surface for mol­ecules I, II, III and IV can be attributed to the different environments of each mol­ecule in the crystalline state.

## Database survey

A search of the Cambridge Structural Database (CSD Version 5.41, update of November 2019; Groom *et al.*, 2016 ▸) for the thieno[2,3-*b*]pyridine moiety yielded ten structures closely related to the title compound: ethyl 3-amino-6-methyl-2-[(4-methyl­phen­yl)carbamo­yl]-4-[(*E*)-2-phenyl­ethen­yl]thieno[2,3-*b*]pyridine-5-carboxyl­ate hydrate (TACXED; Mague *et al.*, 2016*a* ▸), diethyl 3-amino-6-methyl-4-[(*E*)-2-phenyl­ethen­yl]thieno[2,3-*b*]pyridine-2,5-di­carboxyl­ate (MUZXOW; Mague *et al.*, 2016*b* ▸), 4-[(3-fluoro­phen­yl)amino]­thieno[2,3-*b*]pyridine-5-carb­oxy­lic acid (XEBPIF; Pinheiro *et al.*, 2012 ▸), ethyl 3-amino-2-carbamoyl-4-(4-meth­oxy­phen­yl)-6-methyl­thieno[2,3-*b*]pyridine-5-carboxyl­ate dimethyl sulfoxide solvate (AWETIH; Bakhite *et al.*, 2016*a* ▸), ethyl 3-amino-4-(4-chloro­phen­yl)-2-[(4-meth­oxy­phen­yl)carbamo­yl]-6-phenyl­thieno[2,3-*b*]pyridine-5-carboxyl­ate (ULAROQ; Bakhite *et al.*, 2016*b* ▸), ethyl 3-(4-methyl­benzene­sulfonamido)­thieno[2,3-*b*]pyridine-2-carboxyl­ate (GOLDUH; Zhang *et al.*, 2009 ▸), ethyl 3-amino­thieno[2,3-*b*]pyridine-2-carboxyl­ate (QOLPEN; Zheng *et al.*, 2009 ▸), 4-(4-bromo­phen­yl)-2,5-bis­(eth­oxy­carbon­yl)-6-methyl­thieno[2,3-*b*]pyridine (WUVZES; Novoa de Armas *et al.*, 2003 ▸), 5-acetyl-3-amino-4-(4-meth­oxy­phen­yl)-6-methyl­thieno[2,3-*b*]pyridine-2-carbo­nitrile (NEQSUA; Mo­hamed *et al.*, 2017 ▸) and 2-amino-6-benzyl-3-(eth­oxy­carbon­yl)-4,5,6,7-tetra­hydro­thieno[2,3-*c*]pyridin-6-ium (hydrogen bis­(4-meth­oxy­phen­yl)di­phospho­nate) (RUTRUV; Mague *et al.*, 2015 ▸).

In the crystal of TACXED, mutual N—H⋯O hydrogen bonds form dimers, which are then associated into chains parallel to the *c* axis through O—H⋯N hydrogen bonds involving the solvent water mol­ecule. In the crystal of MUZXOW, the bicyclic core of the compound is slightly folded [1.9 (1)°], while pairwise inter­molecular N—H⋯O hydrogen bonding forms dimers across centres of symmetry. In the crystal of XEBPIF, an intra­molecular N—H⋯O_carbon­yl_ hydrogen bond closes an *S*(6) ring. Supra­molecular chains along [01 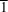 ] mediated by O—H⋯N(pyridine) hydrogen bonds form in the crystal. A three-dimensional network is completed by π–π inter­actions occurring between the benzene ring and the two rings of the thieno[2,3-*b*]pyridine unit. In the crystal of AWETIH, mol­ecules are linked by pairs of N—H⋯O hydrogen bonds, forming inversion dimers with an 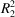 (8) ring motif. Within the dimers, which stack along the *a*-axis direction, there is a weak π–π inter­action involving inversion-related thio­phene rings. In the crystal of ULAROQ, the conformation of the title mol­ecule is partially determined by an intra­molecular N—H⋯O hydrogen bond, forming an *S*(6) loop, and an N—H⋯π inter­action involving the centroid of the 4-chloro­phenyl ring. In the crystal, mol­ecules are linked by pairs of N—H⋯O hydrogen bonds, forming inversion dimers with an 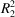 (20) ring motif. In the crystal of GOLDUH, the amino and carbonyl groups are nearly coplanar with the heterocyclic ring system. There are two N—H⋯O hydrogen-bonding inter­actions involving the same N—H donor set and two different acceptors, one in an intra­molecular bond helping to fix the mol­ecular conformation and the other defining a dimeric structure around the symmetry centre at (0, ½, ½). In the crystal of QOLPEN, mol­ecules are linked into a zigzag sheet propagating along the *b-*axis direction by inter­molecular N—H⋯O and N—H⋯N hydrogen bonds. WUVZES crystallizes with two mol­ecules in the asymmetric unit. The crystal structure is stabilized by inter­molecular and intra­molecular C—H⋯O hydrogen bonds. The asymmetric unit of NEQSUA likewise comprises two mol­ecules, which differ primarily in the orientations of the acetyl and *p*-anisyl substituents. In the crystal, N—H⋯O hydrogen bonds form chains extending parallel to (110). The asymmetric unit of the mol­ecular salt RUTRUV comprises two cations and two anions. Each cation features an intra­molecular N—H⋯O hydrogen bond, which closes an *S*(6) ring; in each case the hydro­pyridine ring adopts a half-chair conformation. In the crystal, O—H⋯O and N—H⋯O hydrogen bonds link the components into [100] chains. Numerous C—H⋯O inter­actions cross-link the chains into a three-dimensional network.

## Synthesis and crystallization

To a suspension of 5-acetyl-3-cyano-1,2-di­hydro-6-methyl-4-styryl-2-thioxo­pyridine (2.94 g, 10 mmol), *N*-phenyl-2-chloro­acetamide (1.70 g, 10 mmol) in an ethanol solution (60 ml) was added, together with sodium ethoxide (22 mmol, 0.51 g sodium dissolved in 30 ml absolute ethanol). The resulting mixture was refluxed for 10 minutes. The solid that precipitated after cooling was collected and recrystallized from ethanol to give the title compound in the form of yellow crystals, yield 92%; m.p. 481–483 K. IR (cm^−1^): 3452, 3292, 3220 (NH_2_, NH), 3027 (C—H, aromatic), 1701 (C=O, acet­yl) and 1633 (C=O, anilide). ^1^H NMR: δ 9.59 (*s*, 1H, NH), 7.85–7.88 (*d*, *J* = 15 Hz, 1H, CH=C), 7.07–7.69 (*m*, 10H, Ar—H), 6.79 (*s*, 2H, NH_2_), 6.71–6.74 (*d*, *J* = 15 Hz, 1H, C=CH), 2.52 (*s*, 3H, COCH_3_), δ 2.42 (*s*, 3H, CH_3_ attached to pyridine ring). ^13^C NMR: δ 205.61, 164.34, 158.93, 154.69, 148.61, 140.86, 139.61 (CH of CH=CH), 139.18, 136.00, 133.67, 129.59 (CH), 129.30 (CH), 128.89 (CH), 127.85 (CH), 124.12 (CH), 122.21 (CH of CH=CH), 122.02 (CH), 121.84 (CH), 121.85, 121.25, 98.87, 32.87 (CH_3_ of acetyl group), 23.27 (CH_3_ attached to pyridine ring). MS: *m*/z 427.14 (*M*,^+^ 100%). Analysis calculated for C_25_H_21_N_3_O_2_S (427.13): C 70.24, H 4.95, N 9.84%. Found: C 70.51, H 4.85, N, 9.90%.

## Refinement details

Crystal data, data collection and structure refinement details are summarized in Table 6 ▸. H atoms attached to carbon were placed in calculated positions (C—H = 0.95–0.98 Å) while those attached to nitro­gen were derived from a difference-Fourier map and their parameters adjusted to give N—H = 0.91 Å. All H atoms were included as riding contributions with isotropic displacement parameters 1.2–1.5 times those of the attached atoms.

## Supplementary Material

Crystal structure: contains datablock(s) I, global. DOI: 10.1107/S2056989022000743/wm5633sup1.cif

Structure factors: contains datablock(s) I. DOI: 10.1107/S2056989022000743/wm5633Isup2.hkl

Supporting information file. DOI: 10.1107/S2056989022000743/wm5633Isup3.cml

CCDC reference: 2143707

Additional supporting information:  crystallographic information; 3D view; checkCIF report

## Figures and Tables

**Figure 5:**
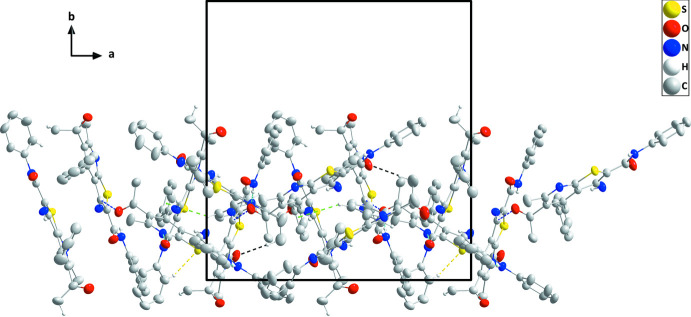
Packing viewed along the *c*-axis direction with inter­molecular inter­actions depicted as in Fig. 2 ▸.

**Table 1:** Selected torsion angles (°)

Mol­ecule I		Mol­ecule III	
C4—C3—C9—C10	126.8 (2)	C54—C53—C59—C60	−57.7 (3)
C9—C10—C11—C12	162.2 (2)	C59—C60—C61—C62	−166.9 (2)
C19—N3—C20—C21	−9.9 (3)	C69—N9—C70—C71	−44.4 (3)
			
Mol­ecule II		Mol­ecule IV	
C29—C28—C34—C35	−51.8 (3)	C79—C78—C84—C85	108.2 (2)
C34—C35—C36—C41	178.4 (2)	C84—C85—C86—C87	145.5 (2)
C44—N6—C45—C50	−23.3 (3)	C94—N12—C95—C100	−25.0 (3)

**Table 2:** Dihedral angles (°)

Planes	Angle
Mol­ecule I	
N1/C1–C5 *vs* C4/C5/S1/C17/C18	2.18 (8)
N1/C1–C5 *vs* C10–C16	70.47 (5)
N1/C1–C5 *vs* C20–C25	12.78 (8)
	
Mol­ecule II	
N4/C26–C30 *vs* C29/C30/S2/C43/C42	4.0 (1)
N4/C26–C30 *vs* C36–C41	47.01 (5)
N4/C26–C30 *vs* C45–C50	27.4 (1)
	
Mol­ecule III	
N7/C51–C55 *vs* C54/C55/S3/C68/C67	3.20 (8)
N7/C51–C55 *vs* C61–C66	48.96 (6)
N7/C51–C55 *vs* C70–C75	35.64 (8)
	
Mol­ecule IV	
N10/C76–C80 *vs* C80/S4/C93/C92	2.4 (1)
N10/C76–C80 *vs* C95–C100	32.11 (8)
N10/C76–C80 *vs* C86–C91	77.15 (6)

**Table 3:** Hydrogen-bond geometry (Å, °) *Cg*8, *Cg*14 and *Cg*18 are the centroids of the C36–C41, C70–C75 and C86–C91 benzene rings, respectively.

*D*—H⋯*A*	*D*—H	H⋯*A*	*D*⋯*A*	*D*—H⋯*A*
N2—H2*A*⋯O2	0.91	1.98	2.703 (2)	135
N3—H3*A*⋯N4^i^	0.91	2.31	3.190 (2)	164
C8—H8*B*⋯*Cg*14	0.98	2.67	3.537 (2)	148
C21—H21⋯O2	0.95	2.22	2.825 (2)	121
N5—H5*A*⋯O4	0.91	2.03	2.717 (2)	131
N6—H6*D*⋯N7^ii^	0.91	2.38	3.231 (2)	157
C33—H33*C*⋯O8^iii^	0.98	2.47	3.411 (3)	162
C41—H41⋯*Cg*18^iii^	0.95	2.94	3.673 (2)	135
C58—H58*B*⋯*Cg*8^iv^	0.98	2.91	3.534 (3)	122
C75—H75⋯S4^v^	0.95	2.87	3.781 (2)	160
N8—H8*D*⋯O6	0.91	1.98	2.701 (2)	135
N9—H9*A*⋯N10^v^	0.91	2.22	3.106 (2)	164
N11—H11*A*⋯O8	0.91	1.99	2.697 (2)	134
N12—H12*A*⋯N1^vi^	0.91	2.30	3.193 (2)	168

Symmetry codes: (i) x+{\script{1\over 2}}, -y+{\script{1\over 2}}, z-{\script{1\over 2}}; (ii) x-{\script{1\over 2}}, -y+{\script{1\over 2}}, z+{\script{1\over 2}}; (iii) x-1, y, z; (iv) -x+{\script{1\over 2}}, y-{\script{1\over 2}}, -z+{\script{1\over 2}}; (v) x-{\script{1\over 2}}, -y+{\script{1\over 2}}, z-{\script{1\over 2}}; (vi) x+{\script{1\over 2}}, -y+{\script{1\over 2}}, z+{\script{1\over 2}}.

**Table 4:** Summary of short inter­atomic contacts (Å) in the title compound

Contact	distance	Symmetry operation
H3*A*⋯N4	2.31	{1\over 2} + *x*, {1\over 2} − *y*, −{1\over 2} + *z*
O1⋯H40	2.64	{1\over 2} − *x*, {1\over 2} + *y*, {1\over 2} − *z*
H13⋯H8*C*	2.38	1 − *x*, 1 − *y*, 1 − *z*
H21⋯H97	2.33	2 − *x*, −*y*, 1 − *z*
N1⋯H12*A*	2.30	−{1\over 2} + *x*, {1\over 2} − *y*, −{1\over 2} + *z*
H2*B*⋯O7	2.62	*x*, *y*, *z*
H2*A*⋯H60	2.55	*x*, *y*, *z*
C5⋯H22	3.03	{3\over 2} − *x*, {1\over 2} + *y*, {1\over 2} − *z*
C8⋯H47	3.09	−*x*, 1 − *y*, 1 − *z*
H15⋯O3	2.71	*x*, *y*, *z*
H24⋯H83*B*	2.58	{3\over 2} − *x*, −{1\over 2} + *y*, {1\over 2} − *z*
H6*C*⋯H87	2.42	{3\over 2} − *x*, {1\over 2} + *y*, {1\over 2} − *z*
H13⋯C48	3.06	1 + *x*, *y*, *z*
H24⋯C50	3.07	{1\over 2} − *x*, −{1\over 2} + *y*, {1\over 2} − *z*
H6*D*⋯N7	2.38	−{1\over 2} + *x*, {1\over 2} − *y*, {1\over 2} + *z*
O3⋯H8*E*	2.55	*x*, *y*, *z*
H33*C*⋯O8	2.47	− 1 + *x*, *y*, *z*
H5*A*⋯H56*C*	2.40	{1\over 2} − *x*, {1\over 2} + *y*, {1\over 2} − *z*
H49⋯C28	3.06	−*x*, 1 − *y*, 1 − *z*
H31*A*⋯H89	2.33	−{1\over 2} + *x*, {1\over 2} − *y*, {1\over 2} + *z*
H33*C*⋯H74	2.42	{1\over 2} − *x*, −{1\over 2} + *y*, {1\over 2} − *z*
H38⋯H81*A*	2.44	−{1\over 2} + *x*, {1\over 2} − *y*, −{1\over 2} + *z*
H47⋯C71	2.95	−*x*, 1 − *y*, 1 − *z*
H9*A*⋯N10	2.22	−{1\over 2} + *x*, {1\over 2} − *y*, −{1\over 2} + *z*
O5⋯H90	2.69	{3\over 2} − *x*, −{1\over 2} + *y*, {1\over 2} − *z*
O5⋯H97	2.75	2 − *x*, −*y*, 1 − *z*
H64⋯O5	2.72	1 − *x*, −*y*, 1 − *z*
C53⋯H72	3.03	{1\over 2} − *x*, −{1\over 2} + *y*, {1\over 2} − *z*
H62⋯O7	2.64	*x*, *y*, *z*
H65⋯C98	2.89	−1 + *x*, *y*, *z*
H99⋯C76	2.90	2 − *x*, −*y*, 1 − *z*

**Table 5:** Percentage contributions of inter­atomic contacts to the Hirshfeld surface for the title compound

Contact	Percentage contribution			
	Mol­ecule I	Mol­ecule II	Mol­ecule III	Mol­ecule IV
H⋯H	46.5	47.0	44.7	45.5
C⋯H/H⋯C	22.7	27.9	28.1	20.2
O⋯H/H⋯O	9.7	8.9	11.3	12.6
N⋯H/H⋯N	5.1	5.5	5.0	6.5
C⋯C	4.9	2.2	1.8	5.6
S⋯H/H⋯S	3.2	2.9	3.3	3.4
O⋯C/C⋯O	2.5	1.4	1.2	0.4
S⋯N/N⋯S	1.5	1.5	1.1	1.1
S⋯C/C⋯S	1.3	0.7	1.1	1.6
S⋯S	1.3	1.2	1.2	1.0
N⋯C/C⋯N	1.1	0.8	1.0	1.6
N⋯N	0.2	0.0	0.1	0.0
S⋯C/C⋯S	0.0	0.0	0.0	0.5

**Table 6:** Experimental details

Crystal data	
Chemical formula	C_25_H_21_N_3_O_2_S
*M*_r_	427.51
Crystal system, space group	Monoclinic, *P*2_1_/*n*
Temperature (K)	150
*a*, *b*, *c* (Å)	18.2782 (5), 19.1455 (6), 24.6978 (7)
β (°)	96.323 (1)
*V* (Å^3^)	8590.3 (4)
*Z*	16
Radiation type	Cu *K*α
μ (mm^−1^)	1.56
Crystal size (mm)	0.43 × 0.35 × 0.13

Data collection	
Diffractometer	Bruker D8 VENTURE PHOTON 100 CMOS
Absorption correction	Multi-scan (*SADABS*; Krause *et al.*, 2015 ▸)
*T*_min_, *T*_max_	0.73, 0.82
No. of measured, independent and observed [*I* > 2σ(*I*)] reflections	65284, 17176, 14297
*R*_int_	0.038
(sin θ/λ)_max_ (Å^−1^)	0.626

Refinement	
*R*[*F* ^2^ > 2σ(*F* ^2^)], *wR*(*F* ^2^), *S*	0.043, 0.114, 1.04
No. of reflections	17176
No. of parameters	1126
H-atom treatment	H-atom parameters constrained
Δρ_max_, Δρ_min_ (e Å^−3^)	0.68, −0.39

Computer programs: *APEX3* and *SAINT* (Bruker, 2016 ▸), *SHELXT* (Sheldrick, 2015*a* ▸), *SHELXL* (Sheldrick, 2015*b* ▸), *DIAMOND* (Brandenburg & Putz, 2012 ▸), *OLEX2* (Dolomanov *et al.*, 2009 ▸) and *publCIF* (Westrip, 2010 ▸).

## supplementary crystallographic information

### Crystal data

**Table:**

C_25_H_21_N_3_O_2_S	*F*(000) = 3584
*M**_r_* = 427.51	*D*_x_ = 1.322 Mg m^−^^3^
Monoclinic, *P*2_1_/*n*	Cu *K*α radiation, λ = 1.54178 Å
*a* = 18.2782 (5) Å	Cell parameters from 9118 reflections
*b* = 19.1455 (6) Å	θ = 4.3–74.6°
*c* = 24.6978 (7) Å	µ = 1.56 mm^−^^1^
β = 96.323 (1)°	*T* = 150 K
*V* = 8590.3 (4) Å^3^	Block, yellow
*Z* = 16	0.43 × 0.35 × 0.13 mm

### Data collection

**Table:**

Bruker D8 VENTURE PHOTON 100 CMOS diffractometer	17176 independent reflections
Radiation source: INCOATEC IµS micro–focus source	14297 reflections with *I* > 2σ(*I*)
Mirror monochromator	*R*_int_ = 0.038
Detector resolution: 10.4167 pixels mm^-1^	θ_max_ = 74.7°, θ_min_ = 2.9°
ω scans	*h* = −22→22
Absorption correction: multi-scan (*SADABS*; Krause *et al.*, 2015)	*k* = −23→22
*T*_min_ = 0.73, *T*_max_ = 0.82	*l* = −29→30
65284 measured reflections	

### Refinement

**Table:**

Refinement on *F*^2^	Secondary atom site location: difference Fourier map
Least-squares matrix: full	Hydrogen site location: mixed
*R*[*F*^2^ > 2σ(*F*^2^)] = 0.043	H-atom parameters constrained
*wR*(*F*^2^) = 0.114	*w* = 1/[σ^2^(*F*_o_^2^) + (0.0485*P*)^2^ + 4.7725*P*] where *P* = (*F*_o_^2^ + 2*F*_c_^2^)/3
*S* = 1.04	(Δ/σ)_max_ = 0.001
17176 reflections	Δρ_max_ = 0.68 e Å^−^^3^
1126 parameters	Δρ_min_ = −0.39 e Å^−^^3^
0 restraints	Extinction correction: *SHELXL* (Sheldrick, 2015*b*), Fc^*^=kFc[1+0.001xFc^2^λ^3^/sin(2θ)]^-1/4^
Primary atom site location: dual	Extinction coefficient: 0.00063 (3)

### Special details

**Table:**

Geometry. All esds (except the esd in the dihedral angle between two l.s. planes) are estimated using the full covariance matrix. The cell esds are taken into account individually in the estimation of esds in distances, angles and torsion angles; correlations between esds in cell parameters are only used when they are defined by crystal symmetry. An approximate (isotropic) treatment of cell esds is used for estimating esds involving l.s. planes.
Refinement. Refinement of F^2^ against ALL reflections. The weighted R-factor wR and goodness of fit S are based on F^2^, conventional R-factors R are based on F, with F set to zero for negative F^2^. The threshold expression of F^2^ > 2sigma(F^2^) is used only for calculating R-factors(gt) etc. and is not relevant to the choice of reflections for refinement. R-factors based on F^2^ are statistically about twice as large as those based on F, and R- factors based on ALL data will be even larger. H-atoms attached to carbon were placed in calculated positions (C—H = 0.95 - 0.98 Å) while those attached to nitrogen were placed in locations derived from a difference map and their parameters adjusted to give N—H = 0.91 Å. All were included as riding contributions with isotropic displacement parameters 1.2 - 1.5 times those of the attached atoms.

### Fractional atomic coordinates and isotropic or equivalent isotropic displacement parameters (Å^2^)

**Table:**

	*x*	*y*	*z*	*U*_iso_*/*U*_eq_	
S1	0.62520 (3)	0.30624 (2)	0.17394 (2)	0.03185 (10)	
O1	0.55039 (8)	0.58307 (7)	0.31695 (6)	0.0421 (3)	
O2	0.64768 (8)	0.14490 (7)	0.27516 (5)	0.0406 (3)	
N1	0.58071 (8)	0.43773 (8)	0.18463 (6)	0.0300 (3)	
N2	0.59334 (9)	0.25628 (8)	0.32483 (6)	0.0324 (3)	
H2A	0.615181	0.213520	0.327535	0.039*	
H2B	0.600892	0.286081	0.353598	0.039*	
N3	0.67707 (8)	0.15460 (8)	0.18797 (6)	0.0320 (3)	
H3A	0.675877	0.183762	0.158792	0.038*	
C1	0.55522 (10)	0.48828 (9)	0.21497 (7)	0.0308 (4)	
C2	0.53820 (10)	0.47654 (9)	0.26873 (7)	0.0300 (4)	
C3	0.54733 (9)	0.41048 (9)	0.29249 (7)	0.0281 (3)	
C4	0.57713 (9)	0.35781 (9)	0.26157 (7)	0.0264 (3)	
C5	0.59141 (9)	0.37510 (9)	0.20844 (7)	0.0279 (3)	
C6	0.54387 (12)	0.55831 (10)	0.18774 (8)	0.0416 (4)	
H6A	0.492551	0.572890	0.188234	0.062*	
H6B	0.555166	0.554896	0.149948	0.062*	
H6C	0.576490	0.592769	0.207279	0.062*	
C7	0.50914 (11)	0.53688 (10)	0.29932 (7)	0.0330 (4)	
C8	0.42847 (11)	0.53794 (11)	0.30383 (9)	0.0414 (4)	
H8A	0.412776	0.491869	0.315429	0.062*	
H8B	0.401883	0.549543	0.268329	0.062*	
H8C	0.417730	0.573094	0.330690	0.062*	
C9	0.52653 (10)	0.39531 (10)	0.34737 (7)	0.0304 (4)	
H9	0.494318	0.357032	0.350899	0.037*	
C10	0.54946 (10)	0.43121 (10)	0.39249 (7)	0.0314 (4)	
H10	0.580706	0.470164	0.388737	0.038*	
C11	0.53033 (10)	0.41539 (10)	0.44731 (7)	0.0324 (4)	
C12	0.57324 (12)	0.44375 (11)	0.49233 (8)	0.0388 (4)	
H12	0.613478	0.473367	0.486868	0.047*	
C13	0.55781 (14)	0.42920 (12)	0.54475 (8)	0.0476 (5)	
H13	0.587936	0.448134	0.575009	0.057*	
C14	0.49904 (15)	0.38746 (12)	0.55313 (8)	0.0509 (6)	
H14	0.488555	0.377599	0.589171	0.061*	
C15	0.45492 (14)	0.35963 (12)	0.50907 (9)	0.0490 (5)	
H15	0.413865	0.331271	0.514885	0.059*	
C16	0.47095 (12)	0.37335 (11)	0.45647 (8)	0.0391 (4)	
H16	0.440982	0.353784	0.426397	0.047*	
C17	0.59831 (9)	0.28651 (9)	0.27486 (7)	0.0269 (3)	
C18	0.62344 (9)	0.25232 (9)	0.23108 (7)	0.0292 (4)	
C19	0.64993 (9)	0.18031 (10)	0.23342 (7)	0.0307 (4)	
C20	0.70776 (10)	0.08771 (10)	0.18143 (7)	0.0314 (4)	
C21	0.72491 (11)	0.04053 (11)	0.22391 (8)	0.0399 (4)	
H21	0.715616	0.052318	0.259885	0.048*	
C22	0.75559 (12)	−0.02373 (11)	0.21350 (9)	0.0426 (5)	
H22	0.767168	−0.055687	0.242608	0.051*	
C23	0.76955 (12)	−0.04196 (11)	0.16166 (9)	0.0442 (5)	
H23	0.790212	−0.086241	0.154953	0.053*	
C24	0.75326 (14)	0.00458 (13)	0.11987 (9)	0.0527 (6)	
H24	0.762728	−0.007587	0.084008	0.063*	
C25	0.72304 (13)	0.06933 (12)	0.12953 (8)	0.0457 (5)	
H25	0.712741	0.101362	0.100306	0.055*	
S2	0.03954 (3)	0.33246 (3)	0.57578 (2)	0.04351 (13)	
O3	0.32420 (9)	0.25977 (11)	0.43995 (7)	0.0637 (5)	
O4	−0.09694 (9)	0.43274 (9)	0.46759 (6)	0.0517 (4)	
N4	0.16909 (9)	0.27160 (10)	0.57169 (6)	0.0387 (4)	
N5	0.01371 (10)	0.37310 (11)	0.41885 (6)	0.0463 (4)	
H5A	−0.020564	0.407740	0.416974	0.056*	
H5B	0.047379	0.373820	0.394320	0.056*	
N6	−0.09656 (9)	0.42275 (9)	0.55974 (6)	0.0385 (4)	
H6D	−0.073302	0.401715	0.589899	0.046*	
C26	0.22238 (10)	0.24755 (11)	0.54371 (7)	0.0369 (4)	
C27	0.21831 (10)	0.25353 (10)	0.48685 (7)	0.0331 (4)	
C28	0.15804 (10)	0.28521 (11)	0.45707 (7)	0.0346 (4)	
C29	0.10163 (10)	0.31114 (10)	0.48631 (7)	0.0298 (4)	
C30	0.11108 (10)	0.30046 (10)	0.54284 (7)	0.0331 (4)	
C31	0.28697 (13)	0.21482 (16)	0.57694 (9)	0.0592 (7)	
H31A	0.288331	0.230208	0.614884	0.089*	
H31B	0.332503	0.228994	0.562430	0.089*	
H31C	0.282312	0.163852	0.575239	0.089*	
C32	0.27920 (11)	0.22264 (13)	0.45800 (7)	0.0431 (5)	
C33	0.28079 (16)	0.14500 (16)	0.45355 (13)	0.0716 (8)	
H33A	0.308692	0.125409	0.486193	0.107*	
H33B	0.304358	0.131671	0.421304	0.107*	
H33C	0.230343	0.126882	0.450211	0.107*	
C34	0.15891 (12)	0.28910 (13)	0.39694 (8)	0.0449 (5)	
H34	0.201552	0.308062	0.383690	0.054*	
C35	0.10510 (12)	0.26828 (12)	0.36074 (8)	0.0422 (5)	
H35	0.059907	0.256078	0.373944	0.051*	
C36	0.10852 (12)	0.26214 (11)	0.30152 (8)	0.0396 (4)	
C37	0.17197 (12)	0.27616 (12)	0.27676 (8)	0.0449 (5)	
H37	0.214360	0.294217	0.297862	0.054*	
C38	0.17354 (12)	0.26388 (14)	0.22140 (8)	0.0514 (6)	
H38	0.216954	0.273184	0.204739	0.062*	
C39	0.11155 (12)	0.23804 (12)	0.19073 (8)	0.0425 (5)	
H39	0.112564	0.228469	0.153073	0.051*	
C40	0.04855 (12)	0.22627 (11)	0.21489 (8)	0.0417 (5)	
H40	0.005492	0.210072	0.193532	0.050*	
C41	0.04721 (12)	0.23769 (11)	0.26968 (9)	0.0423 (5)	
H41	0.003374	0.228602	0.285860	0.051*	
C42	0.03557 (10)	0.35116 (10)	0.47085 (7)	0.0333 (4)	
C43	−0.00274 (10)	0.36595 (10)	0.51460 (7)	0.0338 (4)	
C44	−0.06858 (11)	0.40936 (11)	0.51163 (8)	0.0369 (4)	
C45	−0.15844 (11)	0.46469 (11)	0.56711 (8)	0.0406 (4)	
C46	−0.19320 (13)	0.45263 (16)	0.61369 (9)	0.0573 (6)	
H46	−0.176193	0.416450	0.638244	0.069*	
C47	−0.25276 (14)	0.49358 (18)	0.62416 (11)	0.0707 (8)	
H47	−0.275922	0.485663	0.656211	0.085*	
C48	−0.27856 (14)	0.54561 (16)	0.58849 (12)	0.0647 (7)	
H48	−0.319212	0.573612	0.595885	0.078*	
C49	−0.24488 (13)	0.55661 (13)	0.54207 (12)	0.0570 (6)	
H49	−0.263306	0.591860	0.517132	0.068*	
C50	−0.18451 (12)	0.51725 (11)	0.53093 (10)	0.0473 (5)	
H50	−0.161260	0.526015	0.499054	0.057*	
S3	0.41314 (3)	0.24335 (2)	0.18027 (2)	0.03262 (10)	
O5	0.55494 (9)	−0.03140 (9)	0.31956 (7)	0.0561 (4)	
O6	0.34405 (8)	0.37124 (7)	0.28995 (5)	0.0366 (3)	
N7	0.46504 (9)	0.11366 (8)	0.18421 (6)	0.0331 (3)	
N8	0.38422 (9)	0.24697 (9)	0.33573 (6)	0.0358 (4)	
H8D	0.363500	0.289773	0.338965	0.043*	
H8E	0.390783	0.218293	0.360412	0.043*	
N9	0.33751 (8)	0.38233 (8)	0.19765 (6)	0.0318 (3)	
H9A	0.344208	0.362128	0.165217	0.038*	
C51	0.48351 (10)	0.05531 (10)	0.21191 (7)	0.0337 (4)	
C52	0.47698 (10)	0.04870 (10)	0.26806 (7)	0.0322 (4)	
C53	0.45350 (10)	0.10456 (9)	0.29802 (7)	0.0300 (4)	
C54	0.43342 (9)	0.16649 (9)	0.26909 (7)	0.0270 (3)	
C55	0.44097 (9)	0.16667 (9)	0.21287 (7)	0.0285 (3)	
C56	0.51391 (14)	−0.00244 (12)	0.17965 (9)	0.0495 (5)	
H56A	0.494565	−0.047394	0.190622	0.074*	
H56B	0.567744	−0.002688	0.186584	0.074*	
H56C	0.499232	0.005148	0.140725	0.074*	
C57	0.49572 (11)	−0.02065 (10)	0.29534 (8)	0.0375 (4)	
C58	0.43791 (16)	−0.07515 (13)	0.28970 (14)	0.0694 (8)	
H58A	0.402642	−0.066499	0.316094	0.104*	
H58B	0.460575	−0.121146	0.296565	0.104*	
H58C	0.412261	−0.073868	0.252722	0.104*	
C59	0.44971 (11)	0.09547 (10)	0.35740 (7)	0.0351 (4)	
H59	0.420414	0.058376	0.368735	0.042*	
C60	0.48473 (10)	0.13597 (10)	0.39578 (7)	0.0348 (4)	
H60	0.514138	0.172461	0.383629	0.042*	
C61	0.48267 (10)	0.13014 (11)	0.45512 (7)	0.0351 (4)	
C62	0.51007 (11)	0.18508 (13)	0.48754 (8)	0.0441 (5)	
H62	0.532423	0.223576	0.471510	0.053*	
C63	0.50531 (14)	0.18468 (14)	0.54312 (9)	0.0538 (6)	
H63	0.524141	0.222977	0.564808	0.065*	
C64	0.47365 (14)	0.12944 (14)	0.56708 (8)	0.0526 (6)	
H64	0.469875	0.129730	0.605133	0.063*	
C65	0.44757 (17)	0.07403 (15)	0.53596 (10)	0.0637 (7)	
H65	0.426479	0.035234	0.552525	0.076*	
C66	0.45179 (16)	0.07417 (13)	0.47981 (9)	0.0562 (6)	
H66	0.433295	0.035497	0.458403	0.067*	
C67	0.40131 (9)	0.23191 (9)	0.28491 (7)	0.0275 (3)	
C68	0.38802 (9)	0.27729 (9)	0.24124 (7)	0.0285 (3)	
C69	0.35528 (9)	0.34646 (9)	0.24508 (7)	0.0295 (4)	
C70	0.31164 (10)	0.45239 (10)	0.19555 (7)	0.0326 (4)	
C71	0.25865 (11)	0.47501 (11)	0.22759 (8)	0.0394 (4)	
H71	0.237517	0.443184	0.250946	0.047*	
C72	0.23663 (12)	0.54441 (12)	0.22536 (9)	0.0483 (5)	
H72	0.200382	0.559884	0.247398	0.058*	
C73	0.26680 (14)	0.59137 (12)	0.19145 (10)	0.0528 (6)	
H73	0.251683	0.638872	0.190318	0.063*	
C74	0.31914 (14)	0.56837 (12)	0.15928 (10)	0.0535 (6)	
H74	0.340186	0.600310	0.135956	0.064*	
C75	0.34120 (12)	0.49912 (11)	0.16073 (8)	0.0421 (5)	
H75	0.376527	0.483546	0.137950	0.051*	
S4	0.96809 (2)	0.10829 (3)	0.58282 (2)	0.03352 (11)	
O7	0.66763 (8)	0.24504 (8)	0.44738 (6)	0.0469 (4)	
O8	1.10998 (8)	0.08556 (9)	0.47378 (6)	0.0525 (4)	
N10	0.83433 (8)	0.16708 (9)	0.57761 (6)	0.0320 (3)	
N11	0.99537 (9)	0.16551 (9)	0.43311 (6)	0.0394 (4)	
H11A	1.035436	0.139938	0.426578	0.047*	
H11B	0.958515	0.174776	0.406215	0.047*	
N12	1.11828 (8)	0.04831 (9)	0.56184 (7)	0.0361 (3)	
H12A	1.100106	0.051922	0.594580	0.043*	
C76	0.77995 (10)	0.20337 (11)	0.55000 (7)	0.0338 (4)	
C77	0.78529 (10)	0.22969 (10)	0.49697 (7)	0.0305 (4)	
C78	0.84814 (9)	0.21799 (9)	0.47142 (7)	0.0283 (3)	
C79	0.90435 (9)	0.17761 (9)	0.49958 (7)	0.0280 (3)	
C80	0.89395 (9)	0.15499 (9)	0.55204 (7)	0.0289 (3)	
C81	0.71284 (12)	0.21607 (14)	0.57828 (8)	0.0506 (6)	
H81A	0.714743	0.186665	0.610886	0.076*	
H81B	0.668763	0.204530	0.553632	0.076*	
H81C	0.711063	0.265330	0.588846	0.076*	
C82	0.72317 (10)	0.27253 (11)	0.46837 (7)	0.0341 (4)	
C83	0.73380 (13)	0.34962 (12)	0.46793 (11)	0.0520 (6)	
H83A	0.734134	0.367835	0.505044	0.078*	
H83B	0.693495	0.371266	0.444304	0.078*	
H83C	0.780772	0.360506	0.454169	0.078*	
C84	0.85655 (10)	0.24714 (10)	0.41675 (7)	0.0316 (4)	
H84	0.890110	0.284669	0.414592	0.038*	
C85	0.82008 (10)	0.22411 (10)	0.37049 (7)	0.0323 (4)	
H85	0.783976	0.188765	0.372410	0.039*	
C86	0.83325 (9)	0.25100 (10)	0.31645 (7)	0.0310 (4)	
C87	0.82741 (10)	0.20614 (11)	0.27184 (7)	0.0367 (4)	
H87	0.811175	0.159488	0.275900	0.044*	
C88	0.84504 (11)	0.22879 (13)	0.22153 (8)	0.0438 (5)	
H88	0.842385	0.197287	0.191656	0.053*	
C89	0.86645 (11)	0.29714 (13)	0.21487 (8)	0.0453 (5)	
H89	0.878778	0.312654	0.180487	0.054*	
C90	0.86990 (12)	0.34285 (12)	0.25827 (8)	0.0433 (5)	
H90	0.883050	0.390254	0.253365	0.052*	
C91	0.85428 (11)	0.31998 (11)	0.30899 (8)	0.0381 (4)	
H91	0.857941	0.351528	0.338851	0.046*	
C92	0.97321 (9)	0.15270 (10)	0.48329 (7)	0.0297 (4)	
C93	1.01328 (10)	0.11581 (10)	0.52398 (7)	0.0323 (4)	
C94	1.08387 (10)	0.08226 (11)	0.51783 (8)	0.0356 (4)	
C95	1.18535 (10)	0.01034 (10)	0.56321 (8)	0.0352 (4)	
C96	1.22618 (12)	0.00083 (13)	0.61333 (9)	0.0480 (5)	
H96	1.209688	0.020722	0.645068	0.058*	
C97	1.29099 (12)	−0.03754 (15)	0.61753 (11)	0.0573 (6)	
H97	1.318522	−0.043966	0.652093	0.069*	
C98	1.31539 (12)	−0.06623 (13)	0.57180 (11)	0.0556 (6)	
H98	1.359673	−0.092601	0.574634	0.067*	
C99	1.27543 (13)	−0.05655 (12)	0.52205 (11)	0.0528 (6)	
H99	1.292817	−0.075870	0.490422	0.063*	
C100	1.20983 (12)	−0.01894 (11)	0.51702 (9)	0.0445 (5)	
H100	1.182166	−0.013380	0.482413	0.053*	

### Atomic displacement parameters (Å^2^)

**Table:**

	*U*^11^	*U*^22^	*U*^33^	*U*^12^	*U*^13^	*U*^23^
S1	0.0399 (2)	0.0355 (2)	0.02096 (19)	0.00223 (18)	0.00724 (17)	0.00067 (17)
O1	0.0468 (8)	0.0355 (7)	0.0432 (8)	−0.0021 (6)	0.0021 (6)	−0.0060 (6)
O2	0.0534 (8)	0.0395 (7)	0.0312 (7)	0.0106 (6)	0.0155 (6)	0.0081 (6)
N1	0.0308 (7)	0.0337 (8)	0.0252 (7)	−0.0029 (6)	0.0023 (6)	0.0020 (6)
N2	0.0406 (8)	0.0357 (8)	0.0217 (7)	0.0038 (6)	0.0074 (6)	0.0029 (6)
N3	0.0376 (8)	0.0352 (8)	0.0234 (7)	0.0021 (6)	0.0046 (6)	−0.0009 (6)
C1	0.0312 (8)	0.0328 (9)	0.0280 (9)	−0.0028 (7)	0.0016 (7)	0.0000 (7)
C2	0.0303 (8)	0.0323 (9)	0.0271 (8)	−0.0012 (7)	0.0022 (7)	−0.0018 (7)
C3	0.0273 (8)	0.0331 (9)	0.0237 (8)	−0.0025 (7)	0.0015 (6)	−0.0016 (7)
C4	0.0253 (7)	0.0319 (9)	0.0219 (8)	−0.0028 (6)	0.0019 (6)	−0.0002 (7)
C5	0.0286 (8)	0.0336 (9)	0.0212 (8)	−0.0032 (7)	0.0022 (6)	−0.0002 (7)
C6	0.0510 (11)	0.0356 (10)	0.0385 (11)	0.0007 (9)	0.0067 (9)	0.0068 (8)
C7	0.0407 (10)	0.0325 (9)	0.0258 (8)	0.0034 (8)	0.0034 (7)	0.0020 (7)
C8	0.0413 (10)	0.0442 (11)	0.0393 (11)	0.0052 (9)	0.0072 (8)	−0.0041 (9)
C9	0.0317 (8)	0.0331 (9)	0.0271 (9)	0.0010 (7)	0.0063 (7)	−0.0005 (7)
C10	0.0326 (9)	0.0346 (9)	0.0274 (9)	0.0023 (7)	0.0047 (7)	−0.0010 (7)
C11	0.0381 (9)	0.0340 (9)	0.0260 (9)	0.0078 (7)	0.0073 (7)	−0.0037 (7)
C12	0.0438 (10)	0.0431 (11)	0.0294 (9)	0.0019 (8)	0.0029 (8)	−0.0063 (8)
C13	0.0639 (14)	0.0508 (12)	0.0280 (10)	0.0042 (11)	0.0044 (9)	−0.0082 (9)
C14	0.0785 (16)	0.0492 (12)	0.0273 (10)	0.0048 (11)	0.0155 (10)	−0.0026 (9)
C15	0.0649 (14)	0.0475 (12)	0.0383 (11)	−0.0066 (11)	0.0225 (10)	−0.0030 (9)
C16	0.0481 (11)	0.0415 (10)	0.0290 (9)	−0.0015 (9)	0.0101 (8)	−0.0060 (8)
C17	0.0253 (8)	0.0337 (9)	0.0219 (8)	−0.0019 (7)	0.0035 (6)	0.0011 (7)
C18	0.0297 (8)	0.0352 (9)	0.0230 (8)	0.0002 (7)	0.0038 (7)	0.0005 (7)
C19	0.0290 (8)	0.0376 (9)	0.0260 (8)	0.0006 (7)	0.0049 (7)	−0.0014 (7)
C20	0.0285 (8)	0.0354 (9)	0.0306 (9)	−0.0012 (7)	0.0040 (7)	−0.0051 (7)
C21	0.0466 (11)	0.0403 (10)	0.0328 (10)	0.0062 (9)	0.0043 (8)	−0.0021 (8)
C22	0.0450 (11)	0.0412 (11)	0.0407 (11)	0.0068 (9)	0.0014 (9)	−0.0020 (9)
C23	0.0408 (10)	0.0424 (11)	0.0486 (12)	0.0077 (9)	0.0008 (9)	−0.0101 (9)
C24	0.0654 (14)	0.0561 (13)	0.0375 (11)	0.0179 (11)	0.0094 (10)	−0.0092 (10)
C25	0.0564 (13)	0.0488 (12)	0.0326 (10)	0.0129 (10)	0.0080 (9)	−0.0023 (9)
S2	0.0389 (2)	0.0704 (3)	0.0227 (2)	0.0162 (2)	0.00994 (18)	0.0093 (2)
O3	0.0500 (9)	0.0958 (14)	0.0501 (10)	0.0059 (9)	0.0272 (8)	0.0143 (9)
O4	0.0491 (8)	0.0733 (11)	0.0321 (7)	0.0219 (8)	0.0018 (6)	0.0049 (7)
N4	0.0362 (8)	0.0592 (10)	0.0210 (7)	0.0114 (7)	0.0053 (6)	0.0049 (7)
N5	0.0486 (10)	0.0675 (12)	0.0227 (8)	0.0173 (9)	0.0042 (7)	0.0064 (8)
N6	0.0338 (8)	0.0524 (10)	0.0292 (8)	0.0076 (7)	0.0037 (6)	0.0003 (7)
C26	0.0341 (9)	0.0543 (12)	0.0226 (9)	0.0038 (8)	0.0048 (7)	0.0006 (8)
C27	0.0306 (9)	0.0475 (11)	0.0218 (8)	−0.0023 (8)	0.0052 (7)	−0.0029 (8)
C28	0.0353 (9)	0.0476 (11)	0.0214 (8)	−0.0012 (8)	0.0053 (7)	−0.0007 (8)
C29	0.0309 (8)	0.0378 (9)	0.0204 (8)	−0.0043 (7)	0.0017 (6)	0.0012 (7)
C30	0.0343 (9)	0.0443 (10)	0.0213 (8)	−0.0001 (8)	0.0061 (7)	0.0045 (7)
C31	0.0503 (13)	0.098 (2)	0.0289 (10)	0.0291 (13)	0.0016 (9)	−0.0006 (12)
C32	0.0328 (9)	0.0751 (15)	0.0214 (9)	0.0036 (10)	0.0027 (7)	−0.0046 (9)
C33	0.0606 (15)	0.0814 (19)	0.0758 (19)	0.0055 (14)	0.0199 (14)	−0.0332 (16)
C34	0.0426 (11)	0.0654 (14)	0.0269 (10)	0.0016 (10)	0.0056 (8)	0.0013 (9)
C35	0.0399 (10)	0.0528 (12)	0.0351 (10)	−0.0063 (9)	0.0087 (8)	0.0004 (9)
C36	0.0518 (11)	0.0409 (10)	0.0271 (9)	0.0030 (9)	0.0084 (8)	0.0017 (8)
C37	0.0471 (11)	0.0560 (13)	0.0292 (10)	−0.0133 (10)	−0.0063 (8)	−0.0006 (9)
C38	0.0434 (11)	0.0829 (17)	0.0284 (10)	−0.0175 (11)	0.0064 (9)	0.0038 (10)
C39	0.0470 (11)	0.0585 (13)	0.0229 (9)	−0.0094 (10)	0.0072 (8)	−0.0087 (9)
C40	0.0455 (11)	0.0439 (11)	0.0360 (10)	−0.0078 (9)	0.0055 (9)	−0.0056 (9)
C41	0.0439 (11)	0.0450 (11)	0.0394 (11)	−0.0031 (9)	0.0111 (9)	0.0007 (9)
C42	0.0352 (9)	0.0413 (10)	0.0229 (8)	−0.0020 (8)	0.0014 (7)	0.0022 (7)
C43	0.0335 (9)	0.0441 (10)	0.0239 (8)	0.0007 (8)	0.0035 (7)	0.0033 (8)
C44	0.0355 (9)	0.0466 (11)	0.0284 (9)	0.0022 (8)	0.0025 (7)	0.0009 (8)
C45	0.0335 (9)	0.0486 (11)	0.0392 (11)	0.0012 (8)	0.0010 (8)	−0.0112 (9)
C46	0.0418 (11)	0.0942 (19)	0.0361 (11)	0.0164 (12)	0.0057 (9)	−0.0009 (12)
C47	0.0483 (13)	0.114 (2)	0.0505 (14)	0.0213 (15)	0.0103 (11)	−0.0146 (15)
C48	0.0429 (12)	0.0757 (18)	0.0747 (18)	0.0148 (12)	0.0025 (12)	−0.0252 (15)
C49	0.0482 (13)	0.0444 (12)	0.0765 (17)	0.0051 (10)	−0.0014 (12)	−0.0089 (12)
C50	0.0426 (11)	0.0405 (11)	0.0587 (14)	0.0005 (9)	0.0050 (10)	−0.0040 (10)
S3	0.0428 (2)	0.0355 (2)	0.0206 (2)	0.00549 (18)	0.00827 (17)	0.00122 (17)
O5	0.0571 (10)	0.0566 (10)	0.0516 (9)	0.0090 (8)	−0.0073 (8)	0.0142 (8)
O6	0.0463 (7)	0.0397 (7)	0.0243 (6)	0.0089 (6)	0.0062 (5)	−0.0027 (5)
N7	0.0393 (8)	0.0370 (8)	0.0232 (7)	0.0047 (7)	0.0047 (6)	−0.0041 (6)
N8	0.0488 (9)	0.0388 (8)	0.0212 (7)	0.0105 (7)	0.0103 (6)	0.0022 (6)
N9	0.0373 (8)	0.0353 (8)	0.0232 (7)	0.0046 (6)	0.0054 (6)	−0.0004 (6)
C51	0.0377 (9)	0.0351 (9)	0.0279 (9)	0.0033 (8)	0.0026 (7)	−0.0048 (7)
C52	0.0339 (9)	0.0340 (9)	0.0283 (9)	0.0007 (7)	0.0011 (7)	−0.0012 (7)
C53	0.0310 (8)	0.0338 (9)	0.0251 (8)	−0.0005 (7)	0.0034 (7)	−0.0013 (7)
C54	0.0270 (8)	0.0328 (9)	0.0215 (8)	−0.0017 (7)	0.0043 (6)	−0.0014 (7)
C55	0.0299 (8)	0.0337 (9)	0.0224 (8)	−0.0002 (7)	0.0046 (6)	−0.0010 (7)
C56	0.0684 (15)	0.0452 (12)	0.0352 (11)	0.0163 (11)	0.0065 (10)	−0.0066 (9)
C57	0.0461 (11)	0.0365 (10)	0.0296 (9)	0.0081 (8)	0.0034 (8)	−0.0018 (8)
C58	0.0663 (16)	0.0359 (12)	0.101 (2)	−0.0039 (11)	−0.0135 (15)	0.0129 (13)
C59	0.0426 (10)	0.0353 (9)	0.0284 (9)	0.0024 (8)	0.0079 (8)	0.0039 (8)
C60	0.0344 (9)	0.0421 (10)	0.0280 (9)	0.0003 (8)	0.0042 (7)	0.0037 (8)
C61	0.0337 (9)	0.0462 (11)	0.0257 (9)	0.0084 (8)	0.0045 (7)	0.0056 (8)
C62	0.0403 (10)	0.0596 (13)	0.0320 (10)	−0.0014 (9)	0.0026 (8)	0.0008 (9)
C63	0.0576 (13)	0.0715 (16)	0.0310 (11)	0.0068 (12)	−0.0007 (10)	−0.0078 (11)
C64	0.0636 (14)	0.0712 (16)	0.0236 (10)	0.0213 (12)	0.0074 (9)	0.0065 (10)
C65	0.090 (2)	0.0650 (16)	0.0380 (12)	−0.0024 (14)	0.0155 (13)	0.0159 (12)
C66	0.0854 (18)	0.0502 (13)	0.0332 (11)	−0.0074 (12)	0.0070 (11)	0.0032 (10)
C67	0.0259 (8)	0.0342 (9)	0.0228 (8)	−0.0014 (7)	0.0044 (6)	−0.0018 (7)
C68	0.0302 (8)	0.0338 (9)	0.0220 (8)	0.0005 (7)	0.0052 (6)	−0.0005 (7)
C69	0.0294 (8)	0.0342 (9)	0.0250 (8)	−0.0005 (7)	0.0044 (7)	−0.0008 (7)
C70	0.0325 (9)	0.0361 (9)	0.0285 (9)	0.0041 (7)	0.0001 (7)	0.0006 (7)
C71	0.0346 (9)	0.0466 (11)	0.0374 (10)	0.0082 (8)	0.0062 (8)	0.0043 (9)
C72	0.0448 (11)	0.0546 (13)	0.0459 (12)	0.0200 (10)	0.0064 (9)	0.0010 (10)
C73	0.0570 (13)	0.0415 (12)	0.0590 (14)	0.0154 (10)	0.0025 (11)	0.0054 (10)
C74	0.0601 (14)	0.0430 (12)	0.0582 (14)	0.0066 (10)	0.0106 (11)	0.0160 (11)
C75	0.0462 (11)	0.0441 (11)	0.0373 (10)	0.0062 (9)	0.0105 (9)	0.0064 (9)
S4	0.0305 (2)	0.0476 (3)	0.0227 (2)	0.00580 (18)	0.00392 (16)	0.00587 (18)
O7	0.0366 (7)	0.0569 (9)	0.0453 (8)	0.0054 (7)	−0.0037 (6)	0.0003 (7)
O8	0.0429 (8)	0.0781 (11)	0.0396 (8)	0.0213 (8)	0.0179 (6)	0.0149 (8)
N10	0.0310 (7)	0.0452 (9)	0.0201 (7)	0.0034 (6)	0.0051 (6)	0.0013 (6)
N11	0.0358 (8)	0.0562 (10)	0.0278 (8)	0.0104 (7)	0.0112 (6)	0.0095 (7)
N12	0.0318 (8)	0.0436 (9)	0.0330 (8)	0.0076 (7)	0.0046 (6)	0.0020 (7)
C76	0.0320 (9)	0.0467 (10)	0.0232 (8)	0.0043 (8)	0.0058 (7)	0.0000 (8)
C77	0.0302 (8)	0.0378 (9)	0.0238 (8)	0.0016 (7)	0.0038 (7)	0.0016 (7)
C78	0.0299 (8)	0.0339 (9)	0.0213 (8)	−0.0008 (7)	0.0032 (6)	0.0009 (7)
C79	0.0286 (8)	0.0334 (9)	0.0222 (8)	−0.0007 (7)	0.0040 (6)	0.0002 (7)
C80	0.0289 (8)	0.0368 (9)	0.0211 (8)	−0.0011 (7)	0.0037 (6)	−0.0003 (7)
C81	0.0411 (11)	0.0818 (17)	0.0309 (10)	0.0166 (11)	0.0128 (9)	0.0094 (11)
C82	0.0312 (9)	0.0463 (11)	0.0257 (9)	0.0056 (8)	0.0078 (7)	0.0023 (8)
C83	0.0448 (11)	0.0453 (12)	0.0649 (15)	0.0094 (10)	0.0015 (11)	0.0081 (11)
C84	0.0319 (9)	0.0377 (9)	0.0258 (9)	−0.0001 (7)	0.0048 (7)	0.0062 (7)
C85	0.0316 (9)	0.0386 (10)	0.0272 (9)	0.0016 (7)	0.0057 (7)	0.0054 (7)
C86	0.0258 (8)	0.0427 (10)	0.0242 (8)	0.0019 (7)	0.0012 (6)	0.0043 (7)
C87	0.0340 (9)	0.0468 (11)	0.0284 (9)	−0.0029 (8)	−0.0005 (7)	0.0000 (8)
C88	0.0405 (10)	0.0652 (14)	0.0246 (9)	−0.0039 (10)	−0.0011 (8)	−0.0046 (9)
C89	0.0394 (10)	0.0723 (15)	0.0234 (9)	−0.0108 (10)	0.0003 (8)	0.0090 (9)
C90	0.0438 (11)	0.0530 (12)	0.0321 (10)	−0.0087 (9)	−0.0006 (8)	0.0107 (9)
C91	0.0428 (10)	0.0439 (11)	0.0272 (9)	−0.0020 (8)	0.0019 (8)	0.0029 (8)
C92	0.0300 (8)	0.0356 (9)	0.0244 (8)	−0.0009 (7)	0.0069 (7)	0.0009 (7)
C93	0.0317 (9)	0.0397 (10)	0.0257 (8)	0.0019 (7)	0.0047 (7)	0.0020 (7)
C94	0.0315 (9)	0.0443 (10)	0.0317 (9)	0.0050 (8)	0.0069 (7)	0.0038 (8)
C95	0.0281 (8)	0.0350 (9)	0.0426 (10)	0.0014 (7)	0.0047 (8)	0.0042 (8)
C96	0.0380 (10)	0.0656 (14)	0.0412 (11)	0.0116 (10)	0.0088 (9)	0.0110 (10)
C97	0.0381 (11)	0.0764 (17)	0.0576 (14)	0.0141 (11)	0.0067 (10)	0.0240 (13)
C98	0.0378 (11)	0.0516 (13)	0.0795 (17)	0.0144 (10)	0.0162 (11)	0.0150 (12)
C99	0.0470 (12)	0.0421 (12)	0.0714 (16)	0.0090 (10)	0.0162 (11)	−0.0098 (11)
C100	0.0397 (10)	0.0430 (11)	0.0501 (12)	0.0045 (9)	0.0019 (9)	−0.0110 (9)

### Geometric parameters (Å, º)

**Table:**

S1—C5	1.7209 (18)	S3—C55	1.7241 (18)
S1—C18	1.7516 (18)	S3—C68	1.7471 (17)
O1—C7	1.212 (2)	O5—C57	1.195 (3)
O2—C19	1.238 (2)	O6—C69	1.243 (2)
N1—C1	1.339 (2)	N7—C51	1.334 (2)
N1—C5	1.340 (2)	N7—C55	1.339 (2)
N2—C17	1.375 (2)	N8—C67	1.357 (2)
N2—H2A	0.9100	N8—H8D	0.9100
N2—H2B	0.9099	N8—H8E	0.8193
N3—C19	1.368 (2)	N9—C69	1.366 (2)
N3—C20	1.415 (2)	N9—C70	1.421 (2)
N3—H3A	0.9100	N9—H9A	0.9100
C1—C2	1.414 (2)	C51—C52	1.411 (3)
C1—C6	1.504 (3)	C51—C56	1.505 (3)
C2—C3	1.397 (2)	C52—C53	1.395 (3)
C2—C7	1.509 (2)	C52—C57	1.511 (3)
C3—C4	1.410 (2)	C53—C54	1.412 (2)
C3—C9	1.476 (2)	C53—C59	1.486 (2)
C4—C5	1.405 (2)	C54—C55	1.410 (2)
C4—C17	1.447 (2)	C54—C67	1.455 (2)
C6—H6A	0.9800	C56—H56A	0.9800
C6—H6B	0.9800	C56—H56B	0.9800
C6—H6C	0.9800	C56—H56C	0.9800
C7—C8	1.491 (3)	C57—C58	1.480 (3)
C8—H8A	0.9800	C58—H58A	0.9800
C8—H8B	0.9800	C58—H58B	0.9800
C8—H8C	0.9800	C58—H58C	0.9800
C9—C10	1.337 (3)	C59—C60	1.333 (3)
C9—H9	0.9500	C59—H59	0.9500
C10—C11	1.467 (2)	C60—C61	1.474 (2)
C10—H10	0.9500	C60—H60	0.9500
C11—C16	1.390 (3)	C61—C62	1.382 (3)
C11—C12	1.398 (3)	C61—C66	1.384 (3)
C12—C13	1.383 (3)	C62—C63	1.385 (3)
C12—H12	0.9500	C62—H62	0.9500
C13—C14	1.373 (3)	C63—C64	1.371 (4)
C13—H13	0.9500	C63—H63	0.9500
C14—C15	1.387 (3)	C64—C65	1.365 (4)
C14—H14	0.9500	C64—H64	0.9500
C15—C16	1.388 (3)	C65—C66	1.397 (3)
C15—H15	0.9500	C65—H65	0.9500
C16—H16	0.9500	C66—H66	0.9500
C17—C18	1.385 (2)	C67—C68	1.385 (2)
C18—C19	1.460 (3)	C68—C69	1.461 (2)
C20—C25	1.387 (3)	C70—C71	1.386 (3)
C20—C21	1.394 (3)	C70—C75	1.391 (3)
C21—C22	1.388 (3)	C71—C72	1.388 (3)
C21—H21	0.9500	C71—H71	0.9500
C22—C23	1.378 (3)	C72—C73	1.384 (3)
C22—H22	0.9500	C72—H72	0.9500
C23—C24	1.371 (3)	C73—C74	1.381 (3)
C23—H23	0.9500	C73—H73	0.9500
C24—C25	1.389 (3)	C74—C75	1.385 (3)
C24—H24	0.9500	C74—H74	0.9500
C25—H25	0.9500	C75—H75	0.9500
S2—C30	1.7267 (19)	S4—C80	1.7282 (18)
S2—C43	1.7420 (18)	S4—C93	1.7548 (18)
O3—C32	1.209 (3)	O7—C82	1.208 (2)
O4—C44	1.236 (2)	O8—C94	1.237 (2)
N4—C30	1.331 (2)	N10—C76	1.337 (2)
N4—C26	1.337 (2)	N10—C80	1.339 (2)
N5—C42	1.368 (2)	N11—C92	1.368 (2)
N5—H5A	0.9100	N11—H11A	0.9100
N5—H5B	0.9100	N11—H11B	0.9099
N6—C44	1.369 (2)	N12—C94	1.360 (2)
N6—C45	1.415 (3)	N12—C95	1.422 (2)
N6—H6D	0.9099	N12—H12A	0.9099
C26—C27	1.403 (2)	C76—C77	1.417 (2)
C26—C31	1.499 (3)	C76—C81	1.497 (3)
C27—C28	1.394 (3)	C77—C78	1.388 (2)
C27—C32	1.507 (3)	C77—C82	1.511 (2)
C28—C29	1.412 (3)	C78—C79	1.407 (2)
C28—C34	1.489 (3)	C78—C84	1.485 (2)
C29—C30	1.403 (2)	C79—C80	1.399 (2)
C29—C42	1.445 (3)	C79—C92	1.444 (2)
C31—H31A	0.9800	C81—H81A	0.9800
C31—H31B	0.9800	C81—H81B	0.9800
C31—H31C	0.9800	C81—H81C	0.9800
C32—C33	1.491 (4)	C82—C83	1.489 (3)
C33—H33A	0.9800	C83—H83A	0.9800
C33—H33B	0.9800	C83—H83B	0.9800
C33—H33C	0.9800	C83—H83C	0.9800
C34—C35	1.316 (3)	C84—C85	1.333 (3)
C34—H34	0.9500	C84—H84	0.9500
C35—C36	1.475 (3)	C85—C86	1.475 (2)
C35—H35	0.9500	C85—H85	0.9500
C36—C41	1.378 (3)	C86—C87	1.392 (3)
C36—C37	1.395 (3)	C86—C91	1.393 (3)
C37—C38	1.391 (3)	C87—C88	1.387 (3)
C37—H37	0.9500	C87—H87	0.9500
C38—C39	1.383 (3)	C88—C89	1.381 (3)
C38—H38	0.9500	C88—H88	0.9500
C39—C40	1.373 (3)	C89—C90	1.380 (3)
C39—H39	0.9500	C89—H89	0.9500
C40—C41	1.374 (3)	C90—C91	1.386 (3)
C40—H40	0.9500	C90—H90	0.9500
C41—H41	0.9500	C91—H91	0.9500
C42—C43	1.380 (3)	C92—C93	1.372 (3)
C43—C44	1.458 (3)	C93—C94	1.464 (3)
C45—C46	1.393 (3)	C95—C96	1.385 (3)
C45—C50	1.394 (3)	C95—C100	1.389 (3)
C46—C47	1.389 (3)	C96—C97	1.388 (3)
C46—H46	0.9500	C96—H96	0.9500
C47—C48	1.378 (4)	C97—C98	1.374 (4)
C47—H47	0.9500	C97—H97	0.9500
C48—C49	1.376 (4)	C98—C99	1.371 (4)
C48—H48	0.9500	C98—H98	0.9500
C49—C50	1.389 (3)	C99—C100	1.392 (3)
C49—H49	0.9500	C99—H99	0.9500
C50—H50	0.9500	C100—H100	0.9500
			
C5—S1—C18	90.75 (8)	C55—S3—C68	90.36 (8)
C1—N1—C5	116.36 (15)	C51—N7—C55	116.29 (15)
C17—N2—H2A	111.9	C67—N8—H8D	114.4
C17—N2—H2B	114.7	C67—N8—H8E	121.1
H2A—N2—H2B	118.6	H8D—N8—H8E	124.5
C19—N3—C20	127.14 (16)	C69—N9—C70	123.54 (15)
C19—N3—H3A	116.7	C69—N9—H9A	119.9
C20—N3—H3A	116.1	C70—N9—H9A	116.5
N1—C1—C2	122.45 (16)	N7—C51—C52	122.36 (16)
N1—C1—C6	115.68 (16)	N7—C51—C56	115.55 (16)
C2—C1—C6	121.85 (17)	C52—C51—C56	122.07 (17)
C3—C2—C1	120.65 (16)	C53—C52—C51	121.14 (17)
C3—C2—C7	121.03 (16)	C53—C52—C57	120.33 (16)
C1—C2—C7	118.32 (16)	C51—C52—C57	118.53 (16)
C2—C3—C4	117.10 (15)	C52—C53—C54	116.96 (16)
C2—C3—C9	122.21 (16)	C52—C53—C59	118.97 (16)
C4—C3—C9	120.69 (16)	C54—C53—C59	124.06 (16)
C5—C4—C3	117.37 (16)	C55—C54—C53	116.87 (16)
C5—C4—C17	111.20 (15)	C55—C54—C67	110.39 (15)
C3—C4—C17	131.42 (15)	C53—C54—C67	132.63 (15)
N1—C5—C4	125.97 (16)	N7—C55—C54	126.31 (16)
N1—C5—S1	120.74 (13)	N7—C55—S3	119.71 (13)
C4—C5—S1	113.30 (13)	C54—C55—S3	113.95 (13)
C1—C6—H6A	109.5	C51—C56—H56A	109.5
C1—C6—H6B	109.5	C51—C56—H56B	109.5
H6A—C6—H6B	109.5	H56A—C56—H56B	109.5
C1—C6—H6C	109.5	C51—C56—H56C	109.5
H6A—C6—H6C	109.5	H56A—C56—H56C	109.5
H6B—C6—H6C	109.5	H56B—C56—H56C	109.5
O1—C7—C8	123.00 (18)	O5—C57—C58	121.6 (2)
O1—C7—C2	119.96 (17)	O5—C57—C52	121.51 (19)
C8—C7—C2	116.97 (16)	C58—C57—C52	116.92 (18)
C7—C8—H8A	109.5	C57—C58—H58A	109.5
C7—C8—H8B	109.5	C57—C58—H58B	109.5
H8A—C8—H8B	109.5	H58A—C58—H58B	109.5
C7—C8—H8C	109.5	C57—C58—H58C	109.5
H8A—C8—H8C	109.5	H58A—C58—H58C	109.5
H8B—C8—H8C	109.5	H58B—C58—H58C	109.5
C10—C9—C3	125.12 (17)	C60—C59—C53	124.26 (18)
C10—C9—H9	117.4	C60—C59—H59	117.9
C3—C9—H9	117.4	C53—C59—H59	117.9
C9—C10—C11	125.34 (18)	C59—C60—C61	126.87 (18)
C9—C10—H10	117.3	C59—C60—H60	116.6
C11—C10—H10	117.3	C61—C60—H60	116.6
C16—C11—C12	118.37 (17)	C62—C61—C66	118.26 (18)
C16—C11—C10	122.68 (17)	C62—C61—C60	118.09 (18)
C12—C11—C10	118.96 (18)	C66—C61—C60	123.58 (19)
C13—C12—C11	120.8 (2)	C61—C62—C63	120.8 (2)
C13—C12—H12	119.6	C61—C62—H62	119.6
C11—C12—H12	119.6	C63—C62—H62	119.6
C14—C13—C12	120.1 (2)	C64—C63—C62	120.5 (2)
C14—C13—H13	119.9	C64—C63—H63	119.7
C12—C13—H13	119.9	C62—C63—H63	119.7
C13—C14—C15	120.1 (2)	C65—C64—C63	119.6 (2)
C13—C14—H14	119.9	C65—C64—H64	120.2
C15—C14—H14	119.9	C63—C64—H64	120.2
C14—C15—C16	119.8 (2)	C64—C65—C66	120.2 (2)
C14—C15—H15	120.1	C64—C65—H65	119.9
C16—C15—H15	120.1	C66—C65—H65	119.9
C15—C16—C11	120.75 (19)	C61—C66—C65	120.6 (2)
C15—C16—H16	119.6	C61—C66—H66	119.7
C11—C16—H16	119.6	C65—C66—H66	119.7
N2—C17—C18	124.20 (16)	N8—C67—C68	123.25 (16)
N2—C17—C4	123.94 (15)	N8—C67—C54	124.88 (16)
C18—C17—C4	111.85 (15)	C68—C67—C54	111.85 (15)
C17—C18—C19	123.53 (16)	C67—C68—C69	123.77 (15)
C17—C18—S1	112.81 (13)	C67—C68—S3	113.42 (13)
C19—C18—S1	123.56 (13)	C69—C68—S3	122.80 (13)
O2—C19—N3	122.30 (17)	O6—C69—N9	121.72 (16)
O2—C19—C18	120.57 (16)	O6—C69—C68	120.86 (16)
N3—C19—C18	117.13 (16)	N9—C69—C68	117.42 (15)
C25—C20—C21	118.67 (18)	C71—C70—C75	119.70 (18)
C25—C20—N3	117.25 (17)	C71—C70—N9	121.65 (17)
C21—C20—N3	124.06 (16)	C75—C70—N9	118.64 (17)
C22—C21—C20	119.81 (19)	C70—C71—C72	119.6 (2)
C22—C21—H21	120.1	C70—C71—H71	120.2
C20—C21—H21	120.1	C72—C71—H71	120.2
C23—C22—C21	121.1 (2)	C73—C72—C71	120.9 (2)
C23—C22—H22	119.4	C73—C72—H72	119.6
C21—C22—H22	119.4	C71—C72—H72	119.6
C24—C23—C22	119.2 (2)	C74—C73—C72	119.2 (2)
C24—C23—H23	120.4	C74—C73—H73	120.4
C22—C23—H23	120.4	C72—C73—H73	120.4
C23—C24—C25	120.6 (2)	C73—C74—C75	120.6 (2)
C23—C24—H24	119.7	C73—C74—H74	119.7
C25—C24—H24	119.7	C75—C74—H74	119.7
C20—C25—C24	120.6 (2)	C74—C75—C70	120.0 (2)
C20—C25—H25	119.7	C74—C75—H75	120.0
C24—C25—H25	119.7	C70—C75—H75	120.0
C30—S2—C43	90.49 (9)	C80—S4—C93	90.64 (8)
C30—N4—C26	116.71 (15)	C76—N10—C80	116.58 (15)
C42—N5—H5A	113.6	C92—N11—H11A	112.9
C42—N5—H5B	118.7	C92—N11—H11B	115.3
H5A—N5—H5B	117.9	H11A—N11—H11B	121.4
C44—N6—C45	126.74 (17)	C94—N12—C95	125.78 (16)
C44—N6—H6D	116.3	C94—N12—H12A	119.5
C45—N6—H6D	117.0	C95—N12—H12A	114.6
N4—C26—C27	121.87 (17)	N10—C76—C77	122.25 (16)
N4—C26—C31	115.84 (16)	N10—C76—C81	116.61 (16)
C27—C26—C31	122.28 (17)	C77—C76—C81	121.14 (17)
C28—C27—C26	121.02 (17)	C78—C77—C76	120.50 (16)
C28—C27—C32	120.20 (16)	C78—C77—C82	119.46 (15)
C26—C27—C32	118.74 (17)	C76—C77—C82	120.02 (15)
C27—C28—C29	117.52 (16)	C77—C78—C79	117.36 (15)
C27—C28—C34	116.96 (17)	C77—C78—C84	121.77 (16)
C29—C28—C34	125.52 (17)	C79—C78—C84	120.87 (15)
C30—C29—C28	116.16 (16)	C80—C79—C78	117.50 (15)
C30—C29—C42	110.35 (16)	C80—C79—C92	111.56 (15)
C28—C29—C42	133.38 (16)	C78—C79—C92	130.93 (16)
N4—C30—C29	126.64 (17)	N10—C80—C79	125.75 (16)
N4—C30—S2	119.69 (13)	N10—C80—S4	121.44 (13)
C29—C30—S2	113.61 (14)	C79—C80—S4	112.81 (13)
C26—C31—H31A	109.5	C76—C81—H81A	109.5
C26—C31—H31B	109.5	C76—C81—H81B	109.5
H31A—C31—H31B	109.5	H81A—C81—H81B	109.5
C26—C31—H31C	109.5	C76—C81—H81C	109.5
H31A—C31—H31C	109.5	H81A—C81—H81C	109.5
H31B—C31—H31C	109.5	H81B—C81—H81C	109.5
O3—C32—C33	122.7 (2)	O7—C82—C83	122.19 (18)
O3—C32—C27	120.8 (2)	O7—C82—C77	121.10 (18)
C33—C32—C27	116.6 (2)	C83—C82—C77	116.70 (17)
C32—C33—H33A	109.5	C82—C83—H83A	109.5
C32—C33—H33B	109.5	C82—C83—H83B	109.5
H33A—C33—H33B	109.5	H83A—C83—H83B	109.5
C32—C33—H33C	109.5	C82—C83—H83C	109.5
H33A—C33—H33C	109.5	H83A—C83—H83C	109.5
H33B—C33—H33C	109.5	H83B—C83—H83C	109.5
C35—C34—C28	125.0 (2)	C85—C84—C78	124.27 (17)
C35—C34—H34	117.5	C85—C84—H84	117.9
C28—C34—H34	117.5	C78—C84—H84	117.9
C34—C35—C36	126.0 (2)	C84—C85—C86	122.96 (17)
C34—C35—H35	117.0	C84—C85—H85	118.5
C36—C35—H35	117.0	C86—C85—H85	118.5
C41—C36—C37	118.56 (18)	C87—C86—C91	118.59 (17)
C41—C36—C35	118.17 (19)	C87—C86—C85	119.66 (17)
C37—C36—C35	123.17 (19)	C91—C86—C85	121.70 (17)
C38—C37—C36	120.43 (19)	C88—C87—C86	120.73 (19)
C38—C37—H37	119.8	C88—C87—H87	119.6
C36—C37—H37	119.8	C86—C87—H87	119.6
C39—C38—C37	119.6 (2)	C89—C88—C87	120.0 (2)
C39—C38—H38	120.2	C89—C88—H88	120.0
C37—C38—H38	120.2	C87—C88—H88	120.0
C40—C39—C38	119.76 (18)	C90—C89—C88	119.87 (18)
C40—C39—H39	120.1	C90—C89—H89	120.1
C38—C39—H39	120.1	C88—C89—H89	120.1
C39—C40—C41	120.6 (2)	C89—C90—C91	120.3 (2)
C39—C40—H40	119.7	C89—C90—H90	119.8
C41—C40—H40	119.7	C91—C90—H90	119.8
C40—C41—C36	120.97 (19)	C90—C91—C86	120.42 (19)
C40—C41—H41	119.5	C90—C91—H91	119.8
C36—C41—H41	119.5	C86—C91—H91	119.8
N5—C42—C43	123.42 (18)	N11—C92—C93	124.64 (16)
N5—C42—C29	124.04 (17)	N11—C92—C79	123.19 (16)
C43—C42—C29	112.53 (16)	C93—C92—C79	112.16 (15)
C42—C43—C44	124.40 (17)	C92—C93—C94	123.71 (16)
C42—C43—S2	112.87 (14)	C92—C93—S4	112.75 (13)
C44—C43—S2	122.58 (14)	C94—C93—S4	123.41 (14)
O4—C44—N6	122.39 (18)	O8—C94—N12	122.57 (17)
O4—C44—C43	120.97 (17)	O8—C94—C93	119.92 (17)
N6—C44—C43	116.64 (16)	N12—C94—C93	117.51 (16)
C46—C45—C50	119.6 (2)	C96—C95—C100	119.36 (18)
C46—C45—N6	117.1 (2)	C96—C95—N12	117.80 (18)
C50—C45—N6	123.28 (19)	C100—C95—N12	122.81 (18)
C47—C46—C45	119.9 (3)	C95—C96—C97	120.5 (2)
C47—C46—H46	120.1	C95—C96—H96	119.7
C45—C46—H46	120.1	C97—C96—H96	119.7
C48—C47—C46	120.6 (3)	C98—C97—C96	120.1 (2)
C48—C47—H47	119.7	C98—C97—H97	119.9
C46—C47—H47	119.7	C96—C97—H97	119.9
C49—C48—C47	119.4 (2)	C99—C98—C97	119.6 (2)
C49—C48—H48	120.3	C99—C98—H98	120.2
C47—C48—H48	120.3	C97—C98—H98	120.2
C48—C49—C50	121.3 (3)	C98—C99—C100	121.2 (2)
C48—C49—H49	119.3	C98—C99—H99	119.4
C50—C49—H49	119.3	C100—C99—H99	119.4
C49—C50—C45	119.2 (2)	C95—C100—C99	119.2 (2)
C49—C50—H50	120.4	C95—C100—H100	120.4
C45—C50—H50	120.4	C99—C100—H100	120.4
			
C5—N1—C1—C2	−1.8 (2)	C55—N7—C51—C52	1.2 (3)
C5—N1—C1—C6	179.72 (16)	C55—N7—C51—C56	−177.12 (18)
N1—C1—C2—C3	−0.2 (3)	N7—C51—C52—C53	−2.7 (3)
C6—C1—C2—C3	178.19 (17)	C56—C51—C52—C53	175.46 (19)
N1—C1—C2—C7	−179.46 (16)	N7—C51—C52—C57	177.02 (18)
C6—C1—C2—C7	−1.1 (3)	C56—C51—C52—C57	−4.8 (3)
C1—C2—C3—C4	2.8 (2)	C51—C52—C53—C54	2.9 (3)
C7—C2—C3—C4	−177.93 (15)	C57—C52—C53—C54	−176.84 (16)
C1—C2—C3—C9	−176.73 (16)	C51—C52—C53—C59	−178.19 (17)
C7—C2—C3—C9	2.5 (3)	C57—C52—C53—C59	2.1 (3)
C2—C3—C4—C5	−3.4 (2)	C52—C53—C54—C55	−1.7 (2)
C9—C3—C4—C5	176.23 (15)	C59—C53—C54—C55	179.43 (17)
C2—C3—C4—C17	175.09 (17)	C52—C53—C54—C67	174.11 (17)
C9—C3—C4—C17	−5.3 (3)	C59—C53—C54—C67	−4.7 (3)
C1—N1—C5—C4	1.2 (3)	C51—N7—C55—C54	0.0 (3)
C1—N1—C5—S1	−179.11 (13)	C51—N7—C55—S3	−178.04 (14)
C3—C4—C5—N1	1.5 (3)	C53—C54—C55—N7	0.3 (3)
C17—C4—C5—N1	−177.28 (16)	C67—C54—C55—N7	−176.41 (17)
C3—C4—C5—S1	−178.28 (12)	C53—C54—C55—S3	178.45 (13)
C17—C4—C5—S1	2.97 (18)	C67—C54—C55—S3	1.72 (19)
C18—S1—C5—N1	178.74 (15)	C68—S3—C55—N7	176.86 (15)
C18—S1—C5—C4	−1.50 (14)	C68—S3—C55—C54	−1.41 (14)
C3—C2—C7—O1	106.6 (2)	C53—C52—C57—O5	−83.5 (3)
C1—C2—C7—O1	−74.2 (2)	C51—C52—C57—O5	96.7 (2)
C3—C2—C7—C8	−76.4 (2)	C53—C52—C57—C58	97.7 (2)
C1—C2—C7—C8	102.8 (2)	C51—C52—C57—C58	−82.0 (3)
C2—C3—C9—C10	−53.7 (3)	C52—C53—C59—C60	123.5 (2)
C4—C3—C9—C10	126.8 (2)	C54—C53—C59—C60	−57.7 (3)
C3—C9—C10—C11	−178.51 (17)	C53—C59—C60—C61	179.37 (18)
C9—C10—C11—C16	−18.0 (3)	C59—C60—C61—C62	−166.9 (2)
C9—C10—C11—C12	162.22 (19)	C59—C60—C61—C66	10.0 (3)
C16—C11—C12—C13	1.2 (3)	C66—C61—C62—C63	−1.3 (3)
C10—C11—C12—C13	−178.94 (19)	C60—C61—C62—C63	175.8 (2)
C11—C12—C13—C14	−1.1 (3)	C61—C62—C63—C64	0.4 (3)
C12—C13—C14—C15	0.1 (4)	C62—C63—C64—C65	0.9 (4)
C13—C14—C15—C16	0.9 (4)	C63—C64—C65—C66	−1.3 (4)
C14—C15—C16—C11	−0.7 (3)	C62—C61—C66—C65	1.0 (4)
C12—C11—C16—C15	−0.3 (3)	C60—C61—C66—C65	−176.0 (2)
C10—C11—C16—C15	179.87 (19)	C64—C65—C66—C61	0.3 (4)
C5—C4—C17—N2	177.33 (16)	C55—C54—C67—N8	177.21 (17)
C3—C4—C17—N2	−1.2 (3)	C53—C54—C67—N8	1.2 (3)
C5—C4—C17—C18	−3.3 (2)	C55—C54—C67—C68	−1.1 (2)
C3—C4—C17—C18	178.22 (17)	C53—C54—C67—C68	−177.19 (18)
N2—C17—C18—C19	−2.0 (3)	N8—C67—C68—C69	0.9 (3)
C4—C17—C18—C19	178.62 (16)	C54—C67—C68—C69	179.30 (15)
N2—C17—C18—S1	−178.41 (14)	N8—C67—C68—S3	−178.27 (14)
C4—C17—C18—S1	2.18 (19)	C54—C67—C68—S3	0.13 (19)
C5—S1—C18—C17	−0.43 (14)	C55—S3—C68—C67	0.71 (14)
C5—S1—C18—C19	−176.87 (15)	C55—S3—C68—C69	−178.47 (15)
C20—N3—C19—O2	−2.0 (3)	C70—N9—C69—O6	6.1 (3)
C20—N3—C19—C18	177.75 (16)	C70—N9—C69—C68	−174.07 (16)
C17—C18—C19—O2	3.5 (3)	C67—C68—C69—O6	7.2 (3)
S1—C18—C19—O2	179.52 (14)	S3—C68—C69—O6	−173.69 (14)
C17—C18—C19—N3	−176.32 (16)	C67—C68—C69—N9	−172.62 (16)
S1—C18—C19—N3	−0.3 (2)	S3—C68—C69—N9	6.5 (2)
C19—N3—C20—C25	171.75 (19)	C69—N9—C70—C71	−44.3 (3)
C19—N3—C20—C21	−9.9 (3)	C69—N9—C70—C75	134.98 (19)
C25—C20—C21—C22	−1.0 (3)	C75—C70—C71—C72	−1.2 (3)
N3—C20—C21—C22	−179.36 (18)	N9—C70—C71—C72	178.09 (18)
C20—C21—C22—C23	0.0 (3)	C70—C71—C72—C73	0.1 (3)
C21—C22—C23—C24	0.5 (3)	C71—C72—C73—C74	0.4 (4)
C22—C23—C24—C25	0.0 (4)	C72—C73—C74—C75	0.1 (4)
C21—C20—C25—C24	1.5 (3)	C73—C74—C75—C70	−1.2 (4)
N3—C20—C25—C24	180.0 (2)	C71—C70—C75—C74	1.8 (3)
C23—C24—C25—C20	−1.0 (4)	N9—C70—C75—C74	−177.6 (2)
C30—N4—C26—C27	1.2 (3)	C80—N10—C76—C77	−1.7 (3)
C30—N4—C26—C31	−179.6 (2)	C80—N10—C76—C81	179.18 (19)
N4—C26—C27—C28	0.2 (3)	N10—C76—C77—C78	0.4 (3)
C31—C26—C27—C28	−179.0 (2)	C81—C76—C77—C78	179.5 (2)
N4—C26—C27—C32	−177.7 (2)	N10—C76—C77—C82	−177.89 (18)
C31—C26—C27—C32	3.2 (3)	C81—C76—C77—C82	1.1 (3)
C26—C27—C28—C29	0.2 (3)	C76—C77—C78—C79	1.9 (3)
C32—C27—C28—C29	177.97 (18)	C82—C77—C78—C79	−179.79 (16)
C26—C27—C28—C34	179.91 (19)	C76—C77—C78—C84	−177.65 (17)
C32—C27—C28—C34	−2.3 (3)	C82—C77—C78—C84	0.7 (3)
C27—C28—C29—C30	−1.8 (3)	C77—C78—C79—C80	−2.7 (2)
C34—C28—C29—C30	178.53 (19)	C84—C78—C79—C80	176.80 (16)
C27—C28—C29—C42	174.1 (2)	C77—C78—C79—C92	175.63 (18)
C34—C28—C29—C42	−5.6 (4)	C84—C78—C79—C92	−4.8 (3)
C26—N4—C30—C29	−3.1 (3)	C76—N10—C80—C79	0.8 (3)
C26—N4—C30—S2	179.68 (16)	C76—N10—C80—S4	−179.60 (14)
C28—C29—C30—N4	3.5 (3)	C78—C79—C80—N10	1.5 (3)
C42—C29—C30—N4	−173.31 (19)	C92—C79—C80—N10	−177.16 (17)
C28—C29—C30—S2	−179.21 (14)	C78—C79—C80—S4	−178.14 (13)
C42—C29—C30—S2	4.0 (2)	C92—C79—C80—S4	3.2 (2)
C43—S2—C30—N4	174.17 (18)	C93—S4—C80—N10	178.27 (16)
C43—S2—C30—C29	−3.35 (16)	C93—S4—C80—C79	−2.06 (15)
C28—C27—C32—O3	76.2 (3)	C78—C77—C82—O7	103.9 (2)
C26—C27—C32—O3	−106.0 (2)	C76—C77—C82—O7	−77.8 (2)
C28—C27—C32—C33	−104.3 (2)	C78—C77—C82—C83	−76.7 (2)
C26—C27—C32—C33	73.6 (3)	C76—C77—C82—C83	101.7 (2)
C27—C28—C34—C35	128.5 (2)	C77—C78—C84—C85	−72.2 (3)
C29—C28—C34—C35	−51.8 (3)	C79—C78—C84—C85	108.2 (2)
C28—C34—C35—C36	−170.7 (2)	C78—C84—C85—C86	−175.80 (17)
C34—C35—C36—C41	178.4 (2)	C84—C85—C86—C87	145.53 (19)
C34—C35—C36—C37	2.1 (4)	C84—C85—C86—C91	−32.0 (3)
C41—C36—C37—C38	−1.7 (3)	C91—C86—C87—C88	2.6 (3)
C35—C36—C37—C38	174.6 (2)	C85—C86—C87—C88	−174.99 (18)
C36—C37—C38—C39	0.4 (4)	C86—C87—C88—C89	−2.0 (3)
C37—C38—C39—C40	1.5 (4)	C87—C88—C89—C90	−0.3 (3)
C38—C39—C40—C41	−2.2 (4)	C88—C89—C90—C91	2.1 (3)
C39—C40—C41—C36	1.0 (3)	C89—C90—C91—C86	−1.5 (3)
C37—C36—C41—C40	1.0 (3)	C87—C86—C91—C90	−0.8 (3)
C35—C36—C41—C40	−175.5 (2)	C85—C86—C91—C90	176.71 (18)
C30—C29—C42—N5	176.37 (19)	C80—C79—C92—N11	177.73 (17)
C28—C29—C42—N5	0.3 (3)	C78—C79—C92—N11	−0.7 (3)
C30—C29—C42—C43	−2.6 (2)	C80—C79—C92—C93	−2.9 (2)
C28—C29—C42—C43	−178.7 (2)	C78—C79—C92—C93	178.65 (19)
N5—C42—C43—C44	−3.3 (3)	N11—C92—C93—C94	−3.2 (3)
C29—C42—C43—C44	175.74 (18)	C79—C92—C93—C94	177.48 (17)
N5—C42—C43—S2	−178.83 (16)	N11—C92—C93—S4	−179.27 (16)
C29—C42—C43—S2	0.2 (2)	C79—C92—C93—S4	1.4 (2)
C30—S2—C43—C42	1.76 (16)	C80—S4—C93—C92	0.36 (15)
C30—S2—C43—C44	−173.88 (18)	C80—S4—C93—C94	−175.77 (17)
C45—N6—C44—O4	−1.5 (3)	C95—N12—C94—O8	−2.8 (3)
C45—N6—C44—C43	178.27 (19)	C95—N12—C94—C93	177.28 (18)
C42—C43—C44—O4	4.3 (3)	C92—C93—C94—O8	−1.0 (3)
S2—C43—C44—O4	179.46 (17)	S4—C93—C94—O8	174.71 (17)
C42—C43—C44—N6	−175.42 (19)	C92—C93—C94—N12	178.95 (18)
S2—C43—C44—N6	−0.3 (3)	S4—C93—C94—N12	−5.3 (3)
C44—N6—C45—C46	158.0 (2)	C94—N12—C95—C96	157.0 (2)
C44—N6—C45—C50	−23.3 (3)	C94—N12—C95—C100	−25.0 (3)
C50—C45—C46—C47	−0.9 (4)	C100—C95—C96—C97	0.0 (3)
N6—C45—C46—C47	177.9 (2)	N12—C95—C96—C97	178.1 (2)
C45—C46—C47—C48	0.9 (4)	C95—C96—C97—C98	0.3 (4)
C46—C47—C48—C49	0.2 (4)	C96—C97—C98—C99	0.2 (4)
C47—C48—C49—C50	−1.3 (4)	C97—C98—C99—C100	−0.9 (4)
C48—C49—C50—C45	1.2 (4)	C96—C95—C100—C99	−0.8 (3)
C46—C45—C50—C49	−0.1 (3)	N12—C95—C100—C99	−178.7 (2)
N6—C45—C50—C49	−178.8 (2)	C98—C99—C100—C95	1.2 (4)

### Hydrogen-bond geometry (Å, º)

*Cg*8, *Cg*14 and *Cg*18 are the centroids of the C36–C41, C70–C75 and C86–C91 benzene rings, respectively.

**Table:**

*D*—H···*A*	*D*—H	H···*A*	*D*···*A*	*D*—H···*A*
N2—H2*A*···O2	0.91	1.98	2.703 (2)	135
N3—H3*A*···N4^i^	0.91	2.31	3.190 (2)	164
C8—H8*B*···*Cg*14	0.98	2.67	3.537 (2)	148
C21—H21···O2	0.95	2.22	2.825 (2)	121
N5—H5*A*···O4	0.91	2.03	2.717 (2)	131
N6—H6*D*···N7^ii^	0.91	2.38	3.231 (2)	157
C33—H33*C*···O8^iii^	0.98	2.47	3.411 (3)	162
C41—H41···*Cg*18^iii^	0.95	2.94	3.673 (2)	135
C58—H58*B*···*Cg*8^iv^	0.98	2.91	3.534 (3)	122
C75—H75···S4^v^	0.95	2.87	3.781 (2)	160
N8—H8*D*···O6	0.91	1.98	2.701 (2)	135
N9—H9*A*···N10^v^	0.91	2.22	3.106 (2)	164
N11—H11*A*···O8	0.91	1.99	2.697 (2)	134
N12—H12*A*···N1^vi^	0.91	2.30	3.193 (2)	168

Symmetry codes: (i) *x*+1/2, −*y*+1/2, *z*−1/2; (ii) *x*−1/2, −*y*+1/2, *z*+1/2; (iii) *x*−1, *y*, *z*; (iv) −*x*+1/2, *y*−1/2, −*z*+1/2; (v) *x*−1/2, −*y*+1/2, *z*−1/2; (vi) *x*+1/2, −*y*+1/2, *z*+1/2.

## References

[bb1] Abdel-Rahman, A. E., Bakhite, A. E. & Al-Taifi, E. A. (2003). *Pharmazie*, **58**, 372–377. 10.1002/chin.200339135 12856996

[bb2] Abuelhassan, S., Bakhite, E. A.-G., Abdel–Rahman, A. E. & El–Mahdy, A. F. M. (2021). *J. Heterocycl. Chem.* **58**, 1784–1801.

[bb3] Al–Waleedy, S. A. H., Bakhite, E. A., Abbady, M. S. & Abdu–Allah, H. H. M. (2020). *J. Heterocycl. Chem.* **57**, 2379–2388.

[bb4] Bahekar, R. H., Jain, M. R., Jadav, P. A., Prajapati, V. M., Patel, D. N., Gupta, A. A., Sharma, A., Tom, R., Bandyopadhya, D., Modi, H. & Patel, P. R. (2007). *Bioorg. Med. Chem.* **15**, 6782–6795. 10.1016/j.bmc.2007.08.005 17723306

[bb5] Bakhite, E. A., Kaur, M., Mohamed, S. K., Akkurt, M., Jasinski, J. P. & Albayati, M. R. (2016*a*). *IUCrData*, **1**, x161474.

[bb6] Bakhite, E. A., Mague, J. T., Mohamed, S. K., Akkurt, M. & Al-Taifi, E. A. (2016*b*). *IUCrData*, **1**, x160657.

[bb7] Bakhite, E. A.-G. (2003). *Phosphorus Sulfur Silicon*, **178**, 929–992.

[bb23] Bernardino, A. M. R., da Silva Pinheiro, L. C., Rodrigues, C. R., Loureiro, N. L., Castro, H. C., Lanfredi-Rangel, A., Sabatini-Lopes, J., Borges, J. C., Carvalho, J. M., Romeiro, G. A., Ferreira, F. V., Frugulhetti, I. C. P. P. & Vannier-Santos, M. A. (2006). *Bioorg. Med. Chem.* **14**, 5765–5770. 10.1016/j.bmc.2006.03.013 16781157

[bb8] Brandenburg, K. & Putz, H. (2012). *DIAMOND*, Crystal Impact GbR, Bonn, Germany.

[bb9] Bruker (2016). *APEX3* and *SAINT*. Bruker AXS, Inc., Madison, Wisconsin, USA.

[bb10] Dolomanov, O. V., Bourhis, L. J., Gildea, R. J., Howard, J. A. K. & Puschmann, H. (2009). *J. Appl. Cryst.* **42**, 339–341.

[bb11] Dotsenko, V. V., Buryi, D. S., Lukina, D. Yu. & Krivokolysko, S. G. (2020). *Russ. Chem. Bull.* **69**, 1829–1858.

[bb12] El-Dean, A. M. K., Abd-Ella, A. A., Hassanien, R., El-Sayed, M. E. A. & A. Abdel-Raheem, S. A. (2019). *ACS Omega*, **4**, 8406–8412. 10.1021/acsomega.9b00932 PMC6649056 31459929

[bb13] Eldin, S. M. (1999). *Z. Naturforsch. Teil B*, **54**, 674–680.

[bb14] Groom, C. R., Bruno, I. J., Lightfoot, M. P. & Ward, S. C. (2016). *Acta Cryst.* B**72**, 171–179. 10.1107/S2052520616003954 PMC4822653 27048719

[bb15] Krause, L., Herbst-Irmer, R., Sheldrick, G. M. & Stalke, D. (2015). *J. Appl. Cryst.* **48**, 3–10. 10.1107/S1600576714022985 PMC4453166 26089746

[bb16] Litvinov, V. P., Dotsenko, V. V. & Krivokolysko, S. G. (2005). *Russ. Chem. Bull.* **54**, 864–904.

[bb17] Mague, J. T., Akkurt, M., Mohamed, S. K., Bakhite, E. A. & Albayati, M. R. (2016*a*). *Acta Cryst.* E**72**, 297–299. 10.1107/S2056989016001341 PMC4778809 27006791

[bb18] Mague, J. T., Mohamed, S. K., Akkurt, M., Younes, S. H. H., Ahmed, E. K. & Albayati, M. R. (2015). *Acta Cryst.* E**71**, o997–o998. 10.1107/S2056989015022331 PMC4719942 26870570

[bb19] Mague, J. T., Mohamed, S. K., Akkurt, M., Bakhite, E. A. & Albayati, M. R. (2016*b*). *IUCrData*, **1**, x160270.

[bb20] Mohamed, S. K., Mague, J. T., Akkurt, M., Bakhite, E. A. & Al-Taifi, E. A. (2017). *IUCrData*, **2**, x171700.

[bb21] Novoa de Armas, H., Peeters, O. M., Blaton, N. M., De Ranter, C. J., Suárez Navarro, M., Salfrán Solano, E., Verdecia Reyes, Y. & Ochoa Rodríguez, E. (2003). *Acta Cryst.* E**59**, o384–o386. 10.1107/s0108270100010416 11077293

[bb22] Pinheiro, L. C. S., Bernardino, A. M. R., Wardell, S. M. S. V., Wardell, J. L. & Tiekink, E. R. T. (2012). *Acta Cryst.* E**68**, o2217–o2218. 10.1107/S1600536812027195 PMC3394012 22798877

[bb24] Sheldrick, G. M. (2015*a*). *Acta Cryst.* A**71**, 3–8.

[bb25] Sheldrick, G. M. (2015*b*). *Acta Cryst.* C**71**, 3–8.

[bb26] Turner, M. J., McKinnon, J. J., Wolff, S. K., Grimwood, D. J., Spackman, M. A., Jayatilaka, D. & Spackman, M. A. (2017). *Crystal Explorer17*. University of Western Australia.

[bb27] Westrip, S. P. (2010). *J. Appl. Cryst.* **43**, 920–925.

[bb28] Zeng, X. X., Zheng, R.-L., Zhou, T., He, H.-Y., Liu, J.-Y., Zheng, Y., Tong, A.-P., Xiang, M.-L., Song, X.-R., Yang, S.-Y., Yu, L.-T., Wei, Y.-Q., Zhao, Y.-L. & Yang, L. (2010). *Bioorg. Med. Chem. Lett.* **20**, 6282–6285. 10.1016/j.bmcl.2010.08.088 20846862

[bb29] Zhang, W., Zheng, R., Song, H., Yang, S.-Y. & Yu, L.-T. (2009). *Acta Cryst.* E**65**, o257. 10.1107/S1600536809000269 PMC2968187 21581873

[bb30] Zheng, R., Zhang, W., Yu, L.-T., Yang, S.-Y. & Yang, L. (2009). *Acta Cryst.* E**65**, o9. 10.1107/S1600536808039974 PMC2967861 21581727

